# Validation of a 6-Dye Short Tandem Repeat System: A Dry Kit With Lyophilized Amplification Reagent

**DOI:** 10.3389/fgene.2021.705819

**Published:** 2021-09-06

**Authors:** Shuanglin Li, Jinfeng Lin, Honglei Hao, Haiying Jin, Danlu Song, Bofeng Zhu

**Affiliations:** ^1^Department of Forensic Genetics, Multi-Omics Innovative Research Center of Forensic Identification, School of Forensic Medicine, Southern Medical University, Guangzhou, China; ^2^School of Medicine, Ningbo University, Ningbo, China; ^3^Zhejiang Key Laboratory of Forensic Science and Technology, Hangzhou, China; ^4^Ningbo Health Gene Technologies Co., Ltd, Ningbo, China; ^5^Key Laboratory of Shaanxi Province for Craniofacial Precision Medicine Research, College of Stomatology, Xi’an Jiaotong University, Xi’an, China

**Keywords:** forensic science, multiplex-short tandem repeat system, SureID®S6 system, lyophilized PCR reagent, validation

## Abstract

The SureID®S6 system used a lyophilized pellet as the amplification reagent to enable multiplexing of sex-determining marker Amelogenin, 21 autosomal short tandem repeats (STRs), and one Y-STR. To assess the performance, reliability, and limitation of the dry amplification system, the validation studies including PCR condition, reproducibility, sizing and precision, analytical threshold calculation, sensitivity and stochastic threshold calculation, species specificity, stability, mixture, case sample, and population and concordance were conducted according to the Scientific Working Group on DNA Analysis Methods (SWGDAM) Validation Guidelines. Experimental data suggested that the optimal range of total input DNA was from 125 to 500 pg; the appropriate analytical threshold was 80 relative fluorescence units (RFUs) while the stochastic threshold was 260 RFUs; for the stability studies, SureID®S6 system could resist against less than 500 μmol/L of hematin, 100 ng/μl of humic acid, 4 mM of indigotin, 800 mM of tannic acid, and 800 mM of calcium ion. Population and concordance studies using 500 unrelated individuals showed that the combined probability of discrimination (CPD) and cumulative probability of exclusion (CPE) values were 0.999999999999 and 0.999999998416, respectively. The genotypes for the same sample were concordant with the previously validated HUAXIA™ Platinum kit. The validation results demonstrated that the SureID®S6 system could be used for forensic applifications.

## Introduction

PCR is widely used to amplify molecular markers, such as short tandem repeat (STR), single nucleotide polymorphism (SNP), insertion-deletion (InDel) and other molecular markers from forensic samples to solve problems, such as paternity testing, individual identification (Hammond et al., 1994), ancestry inference (Guo et al., 2020), age estimation (Marquez-Ruiz et al., 2020), body fluids identification (Ingold et al., 2020), phenotypic inference (Chaitanya et al., 2018), complicated kinship inference (Kling and Tillmar, 2019), and post-mortem interval estimation (Tu et al., 2018). Traditional PCR reagents are in liquid form and stored at −20°C. Since repeated freezing and thawing may decrease the enzyme activity, and the operation may also increase the possibility of sporadic contamination, these reagents are usually divided into aliquots before storage (Kwok and Higuchi, 1989). To simplify the complex procedures, we developed a new 6-dye fluorescent STR system SureID®S6 (S6), which aimed to simplify PCR procedures by using a lyophilized amplification reagent. The dried reagent mixture of the system is packaged in a separate reaction tube and does not need to be divided into aliquots again. Working with the new system, operators will no longer need to prepare PCR premixtures, and the incidence of sporadic contamination (de Lomas et al., 1992) will decrease at the same time. Apart from the above, advantages of lyophilized reagents include a longer shelf life, lower storage conditions, and ease of transportation.

At present, lyophilized reagents are often applied in pathogen diagnosis due to their portable, field-friendly characteristics. Recent outbreaks of Ebola, COVID-19s, and many other diseases have highlighted the need for such reagents in recent years (Tomaszewicz Brown et al., 2020). The lyophilized reagents applied in pathogen diagnosis had given some tips on developing such a ready-to-use forensic kit. The newly established 6-dye S6 system was designed involving the 23 commonly used loci. To improve the success rate of profiling forensic biological samples from crime scenes, especially the degraded ones, the amplicon sizes were less than 400 bp by placing the STR primers adjacent to the repeat motifs. Among them, 14 STR loci were defined as miniSTRs with the amplified fragment sizes under 250 bp. Compared with the classic liquid reagent system, such as the widely used GlobalFiler™ Internal Quality Control (IQC) PCR Amplification kit (<460 bp, including 10 miniSTRs), more miniSTRs (Parsons et al., 2007) should theoretically help maximize information recovery from degraded samples. Moreover, the S6 system was compatible with direct PCR amplification, and it was devised with a pair of IQC to ensure typing accuracy.

The SureID®S6 PCR amplification system, as a forensic STR kit with lyophilized reagent, is designed to optimize the experimental process and provide the reagents amenable to any forensic labs. Given the characteristics of stability and user friendly, the system is expected to have a wide application in the forensic community. The study aims to verify its effectiveness in forensic DNA laboratories. The SWGDAM Validation Guidelines for DNA Analysis Methods^1^ is followed in the establishment and validation of the system.

## Materials and Methods

### DNA Samples and Experimental Design

#### Establishment of the Lyophilized 6-Dye Multiplex Amplification System and PCR Mix Preparation Using Ready-To-Use Freeze-Dried Reagents

The newly established 6-dye system enables us to simultaneously type the Amelogenin, DYS391, and 21 autosomal STR loci. These loci were selected according to the Combined DNA Index System (CODIS) Core Loci Working Group’s recommendation. The DNA sequences were obtained from GenBank. Primer Premier 5.0 and Oligo 6.44 were applied in primer design for all loci. AutoDimer software was applied to evaluate possible interactions between primers. The final multiplex system was built through a series of experiments. The amplicons for Amelogenin, D8S1179, D21S11, D18S51, and D2S1338 were in the blue channel, and FAM fluorescence material was used for labeling; the amplified products for D2S441, D5S818, D7S820, D6S1043, and Penta D in the green channel were labeled with HEX; D3S1358, TH01, D19S433, D12S391, and DYS391 were labeled with TAMRA and displayed in yellow; the red channel including TPOX, D16S539, D13S317, and FGA were labeled with ROX; the purple channel included VIG fluorescein-labeled STR amplicons for CSF1PO, vWA, D1S1656, and Penta E loci (Supplementary Table S1; Supplementary Figure S1); Internal lane standard (ILS), which contained standard DNA fragments of different sizes, displayed orange fluorescence by labeling SIZE-500(S). A pair of IQCs were placed in the blue channel: the fragment size of the small one was 74 bp while the large one was 430 bp. The fragments of alleles were cloned into pMD18-T, amplified, balanced, and purified to prepare allelic ladders of all loci.

Primer mix, reaction mix, Taq-polymerase, and special substance-stabilizers at predefined optimal concentrations were the main components of the lyophilized reagents. The ratio of magnesium ion and DNA Taq polymerase was adjusted to get a satisfactory performance. With the S6 system, the step of premixture preparing was deleted, the operator only needs to add a total of 10 μl of DNA template and dilution buffer to the tube containing the lyophilized pellet. Then, the PCR can proceed as usual (Supplementary Figure S2).

#### PCR Condition Studies

The PCR condition studies included the tests of cycling number, annealing temperature, reaction volume, and final extension time. The standard PCR parameters remained unchanged in the following tests unless noted otherwise.

For cycling number tests, genomic DNA 9948 (500 pg) was amplified with 2+25, 2+26, 2+27, 2+28, and 2+29 cycles, respectively. Besides, 1,000 pg genomic DNA 9948 was amplified with 2+25, 2+26, and 2+27 cycles; 62.5 pg genomic DNA 9948 was amplified with 2+27, 2+28, and 2+29 cycles, respectively. Each cycle number was tested in triple.

For annealing temperature tests, the parameter series were set in the increment of 1°C: 56, 57, 58, 59, 60, and 61°C. The amplification of each annealing temperature condition was repeated three times by using 500 pg genomic DNA 9948.

Reaction volume tests were conducted using a series of volumes: 8.0, 9.0, 10.0, 11.0, and 12.0 μl. Three blood samples were extracted by the Chelex-100 method, three blood filter papers with one 1.0 mm punch, and 500 pg genomic DNA 9948 were applied in the tests.

The seven samples applied in reaction volume tests were also adopted in the final extension time tests. The final hold step was performed for 5, 10, 15, and 30 min, respectively.

#### Reproducibility Studies

Two male DNA samples with high heterozygosity and genomic DNA 9948 were amplified by using GeneAmp™ PCR System 9700 (Thermo Fisher Scientific, United States), Veriti™ 96-Well Fast Thermal Cycler (Thermo Fisher Scientific, United States), and Eppendorf MasterCycler® nexus GSX1 (Eppendorf, Germany). The two blood samples were obtained from 1.0 mm punches. The concentration of genomic DNA 9948 was 0.5 ng/μl. According to the manufacturer’s recommendation, two operators implemented the experiments at the same time.

#### Sizing and Precision Studies

Twenty-four allelic ladders for 24 capillaries and 23 extracted human genomic DNA samples in 3500xL were used to test the precision of the size calling and the accuracy of allele designation. Loading mixtures for capillary electrophoresis (CE) were made of 8.75 μl of the Hi-Di™ Formamide (Thermo Fisher Scientific, United States) and 0.25 μl size standard solution SIZE-500(S), and then 1.00 μl of the PCR product or allelic ladder was added to the corresponding well on the CE plate. The standard deviation (SD) of the amplicon size of each allele of the ladder was used to evaluate size-calling precision. The amplicons of 23 samples were used to observe whether alleles were yielded at the corresponding position designated by the allelic ladder.

#### Stutter Calculation

A total of 254 DNA profiles of directly amplified blood samples were used to characterize the stutter peaks of 21 autosomal STRs. These samples, a subset of 500 unrelated Zhejiang Han individuals, were tested in population and concordance studies.

#### Analytical Threshold Calculation

The typing profiles of 24 negative controls and the RFUs of peaks generated with the threshold of 1 RFU were used to evaluate the analytical threshold.

#### Sensitivity Study and Stochastic Threshold Calculation

This study was conducted using the male genomic DNA 9948. Before adding into PCR solution, 9948 was serially diluted in a 2-fold decrease to obtain the following amounts of DNA templates: 1 ng (1,000 pg), 500 pg, 250 pg, 125 pg, 62.5 pg, and 31.25 pg. Each concentration was tested three times.

#### Species Specificity Studies

Thirteen samples from domestic or experiment-associated species were collected to test the species specificity. The amount of DNA template was set as follows: 1 ng for 9948; 10 ng each for horse, cow, pig, sheep, dog, cat, chicken, duck, rabbit, mouse, fish, and *Escherichia coli* (JM109). The Qubit® 3.0 Fluorometer (Thermo Fisher Scientific, United States) was used to determine the quantity of each template with the Qubit® dsDNA HS Assay Kit according to the manual. Negative control was contained in the study as well.

#### Stability Studies

Five common inhibitors (hematin, humic acid, indigotin, tannic acid, and calcium ion) were applied to test the stability of the system. The concentrations of inhibitors were set as follows: 0, 200, 500, 800, and 1,500 μmol/L of hematin; 0, 100, 150, 200, 250, 300, and 350 ng/μl of humic acid; 0, 4, 7, 10, and 15 mM of indigotin; 0, 800, 1,000, 1,500, and 2,500 mM of tannic acid; and 0, 3, 10, and 12 mM of calcium ion. Inhibitors were tested in parallel with PowerPlex®21system (PP21, Promega Corporation, United States) and VeriFiler™ Plus Amplification kit (VFP, Thermo Fisher Scientific, United States). A total of 500 pg genomic DNA 9948 was applied in each PCR.

One microliter of genomic DNA 9948 (0.5 ng/μl) was exposed to 28w of WD-9430C Ultraviolet instrument (254 nm) for 0, 2.0, 4.0, and 6.0 h at room temperature to prepare degradation samples.

One microliter of control DNA 9948 (0.5 ng/μl) was used to test the stability of the reagent when it was stored at room temperature or −20°C for 26 months. Then, the reagent was repeatedly frozen and thawed 10 times for the same purpose.

#### Mixture Studies

Two sets of DNA mixtures were prepared in the mixture studies. The first set was male/female mixtures, which were made by mixing up male 9948 with female 9947A at the ratios of 1:1, 1:4, 1:9, 1:19, 19:1, 9:1, and 4:1. The total concentration of the formulated samples was held constantly at 1 ng in 10 μl volume. Each ratio was tested three times. The second set was male/female/male mixtures, consisting of 007, 9947A, and 9948. The ratio was 1:1:8 with a total quantity of 1 ng.

#### Case Samples and Heterozygous Balance Studies

Twenty-four blood specimens, 21 buccal swabs, and 20 samples with epithelial cell abrasions from crime scenes, 11 semen samples, seven costal cartilage bones, one tooth, and three fragments of bones were tested. Chelex-100, ML-DNA Extraction Kit (Bokun Biotech, China), QIAamp DNA Micro Kit (QIAGEN, Germany), and OptiPure Blood DNA (61E; TANBead, China) were used for DNA extraction, a disk (1.0 mm for the filter paper, 0.5 mm for the buccal-indicating FTA® card, and 0.5 mm for the blood FTA® card) taken from filter paper or FTA card were used for direct amplification.

All these samples were used to evaluate the adaptability of the S6 system for multiple sample types by observing the peak morphology and calculating the intra-locus balance.

#### Population and Concordance Studies

Five hundred unrelated Han individuals (no intermarriage within three generations) from Zhejiang province with informed consents were employed in the study. The samples were collected with the consents of the Ethical Committee of Xi’an Jiaotong University and South Medical University, China. A 1.00 mm diameter punch from each blood filter paper was amplified directly. S6 System and HUAXIA™ Platinum Kits (Thermo Fisher Scientific, United States) were used to evaluate the concordance.

### Amplification

Thermal cycling was performed in a 0.2 ml eight-strip tube with the attached cap for PCR (USA Scientific, United States). A lyophilized amplification reagent pellet was at the bottom of each tube. When the extracted DNA was added to each tube, the pellet would be melted in a few seconds. The dilution buffer was used to keep the PCR volume at 10 μl.

The ABI GeneAmp® PCR System 9700 Thermal Cycler (Thermo Fisher Scientific, United States) was employed to perform the amplification with the ramping mode being set to “9,600 emulation mode.” The standard thermal cycling parameters were set to initial incubation at 95°C for 5 min; two cycles consisting of denaturation phase at 94°C for 10 s and annealing/extension phase at 63°C for 90 s, followed by 26~28 cycles of 94°C for 10 s and 59°C for 90 s; and final extension phase was set to 60°C for 15 min. The amplified products were then soaked at 4°C until they were removed. The whole process of PCR needed approximately 70 min.

### Electrophoresis and Analysis

Loading mixtures for CE were made of 8.75 μl of the Hi-Di™ Formamide (Thermo Fisher Scientific, United States) and 0.25 μl size standard solution SIZE-500(S), and then 1.00 μl of the PCR product or allelic ladder was added to the corresponding well on the CE plate. When the plate preparation was done, it was denatured at 95°C for 3 min and then immediately quenched at 0°C for 3 min. CE was performed on Applied Biosystems®3500 or 3500xL (24-capillary) Genetic Analyzer using 36 cm capillary arrays. POP-4™ Polymer (Thermo Fisher Scientific, United States) was applied as the medium. A standard run condition was set as follows: samples injection for 15 s at 1.2 kV; electrophoresis for 24 s at 1.2 kV; and run at 60°C and lasted for 1,210 s.

### Statistical Analysis

Raw data were analyzed by GeneMapper® ID-X (GMID-X) Software v1.2 (Thermo Fisher), and peak height data was analyzed by Excel 2010.

The analytical threshold was calculated using three methods: (1) average peak height plus three times SDs; (2) average peak height plus 10 times SDs; and (3) Y_max_ minus Y_min_, then multiplied 2. Y_max_ represented the highest peak and Y_min_ represented the lowest (Martin et al., 2014).

The stochastic threshold was calculated to avoid the surviving false homozygous peak. It was determined by examining heterozygous loci where one sister allele has dropped below the analytical threshold. Compute the average peak height of the observed false homozygotes and the SD of the RFUs. The average peak height plus three SDs would be the stochastic threshold (Promega Corporation, Internal Validation Guide of Autosomal STR Systems for Forensic Laboratories, 2013).^2^

The intra-locus fluorescence balance was revealed through diving lower peak height by higher peak height within one locus.

Forensic parameters, such as typical paternity index (TPI), polymorphism information content (PIC), heterozygosity (H), power of exclusion (PE), and power of discrimination (PD) were calculated by Modified – powerstate – 5 Software. The cumulative probability of paternity exclusion (CPE) as well as combined probability of discrimination (CPD) were conducted based on the two formulas: CPD = 1−(1−PD_1_) (1−PD_2_)…(1−PD*_k_*), and CPE = 1−(1−PE_1_) (1−PE_2_)…(1−PE*_k_*).

## Results and Discussion

### PCR Condition Studies

In the PCR condition studies, a series of studies were conducted to find optimal PCR amplification parameters. Criteria, such as allele count, average peak height, and heterozygous balance, were used to evaluate the typing results.

For 500 pg input DNA, the recommended cycle number was 2+27 cycles (Supplementary Figure S3). Experimental results showed that allele dropouts were observed at D12S391 and Penta D at cycle number 2+25. Complete profiles were obtained from 2+26 to 2+29 cycles with the average heterozygous peak heights of 3,218, 8,386, 16,357, and 29,047 RFUs, respectively. With the increment of cycle numbers, the number of off-scale peaks also increased. At the optimal cycle number 2+27, the profiles led to a maximum number of alleles displaying heterozygous peak heights between 3,000 and 12,000 RFUs and the minimal occurrence of off-scale peaks.

Furthermore, we explored the performance when input DNA was 1 ng or 62.5 pg. For 1.0 ng input 9948, full profiles could be obtained from 2+25 to 2+27 cycles, but a large area of saturation was observed at 2+27 cycles due to the DNA quantity for CE was overloaded. For 62.5 pg input 9948, no dropout was detected from 2+27 to 2+29 cycles, but the peak height was relatively low at 2+27 cycles, and the peak height ratio (PHR) of several loci fall behind 0.6 due to the DNA quantity for CE was insufficient (Supplementary Figure S4). Since the DNA quantity of forensic samples is variable, if it was higher than 1 ng or lower than 62.5 pg, the cycle number could be slightly adjusted to generate a more acceptable profile.

**Figure 1:**
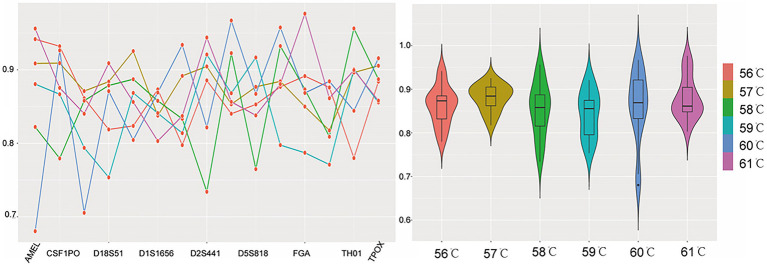
The peak height ratio (PHR) performance when annealing temperature was set at 56, 57, 58, 59, 60, and 61°C. Each temperature was represented by a corresponding color. For the line chart on the left, the *x*-axis listed the loci, and the *y*-axis was the PHR. The red dots of the line chart represented PHR values of locus AMEL, CSF1PO, D12S391, D18S51, D19S433, D1S1656, D21S11, D2S441, D3S1358, D5S818, D8S1179, FGA, Penta D, TH01, and TPOX, respectively. For the violin plot on the right, the *y*-axis still was PHR, while the *x*-axis was the annealing temperature. The violin plot showed the distributions of these PHR values when different annealing temperatures were tested.

For annealing temperature, it was set at 56, 57, 58, 59 (recommended), 60, and 61°C. Within the studied temperature scope, the acceptable peak heights and PHRs of heterozygous loci were produced at all conditions. The average peak heights were 6,478, 8,891, 6,515, 7,514, 7,620, and 8,853 RFUs, respectively (Supplementary Figure S5), the relatively lower peak heights were found at 56 and 58°C. At 60 and 61°C, the distributions of the PHR values were uneven. The qualified peak heights and PHR values (>0.7) were yielded at 57 and 59°C. Since higher annealing temperature could reduce nonspecific amplification, the optimal annealing temperature was 59°C (Figure 1).

The reaction volumes of 8.0, 9.0, 10.0 (recommended), 11.0, and 12.0 μl were tested separately in the reaction volume study. A robust and reliable forensic system should be tolerant of volume fluctuations to some extent, as pipetting errors were usually inevitable in experiments. The results showed that the genotyping accuracy of STR profiles was not affected by volume fluctuations in this range. Most loci showed normal peak morphologies when using different samples (three extracted genomic DNA and three directly amplified blood filter papers; Supplementary Figure S6), but the PHRs of some loci were not ideal when the volume was decreased to 8.0 μl. Since pipetting error hardly exceeds 2.0 μl, the moderately volume change is tolerable when using the S6 system.

The final extension time of 5, 10, 15 (recommended), and 30 min was tested in the study. Sufficient extension time could ensure good peak morphology since Taq-polymerase tends to add extra adenosine at the 3' ends of DNA strands (Magnuson et al., 1996). For 9948, no minus-A peak was detected. For three extracted DNA, normal peak morphologies were obtained at most loci under all conditions, but CSF1PO, D8S1179, and vWA loci had small shoulder peaks for the 5-min-hold (Supplementary Figure S7). For three blood filter papers, nine loci showed minus-A peaks for the 5-min-hold, four loci (D8S1179, TPOX, CSF1PO, and vWA) showed minus-A peaks for the 10-min-hold. Complete terminal nucleotide addition was obtained for 15 and 30 min (Supplementary Figure S8), and thus, the optimal final extension time was 15 min.

### Reproducibility Studies

The genotypes obtained by 9948 and two male DNA samples were concordant across three different PCR instruments and two operators (Supplementary Figure S9). And no significant difference in intra-locus balance or peak height was observed.

### Sizing and Precision Studies

**Figure 2:**
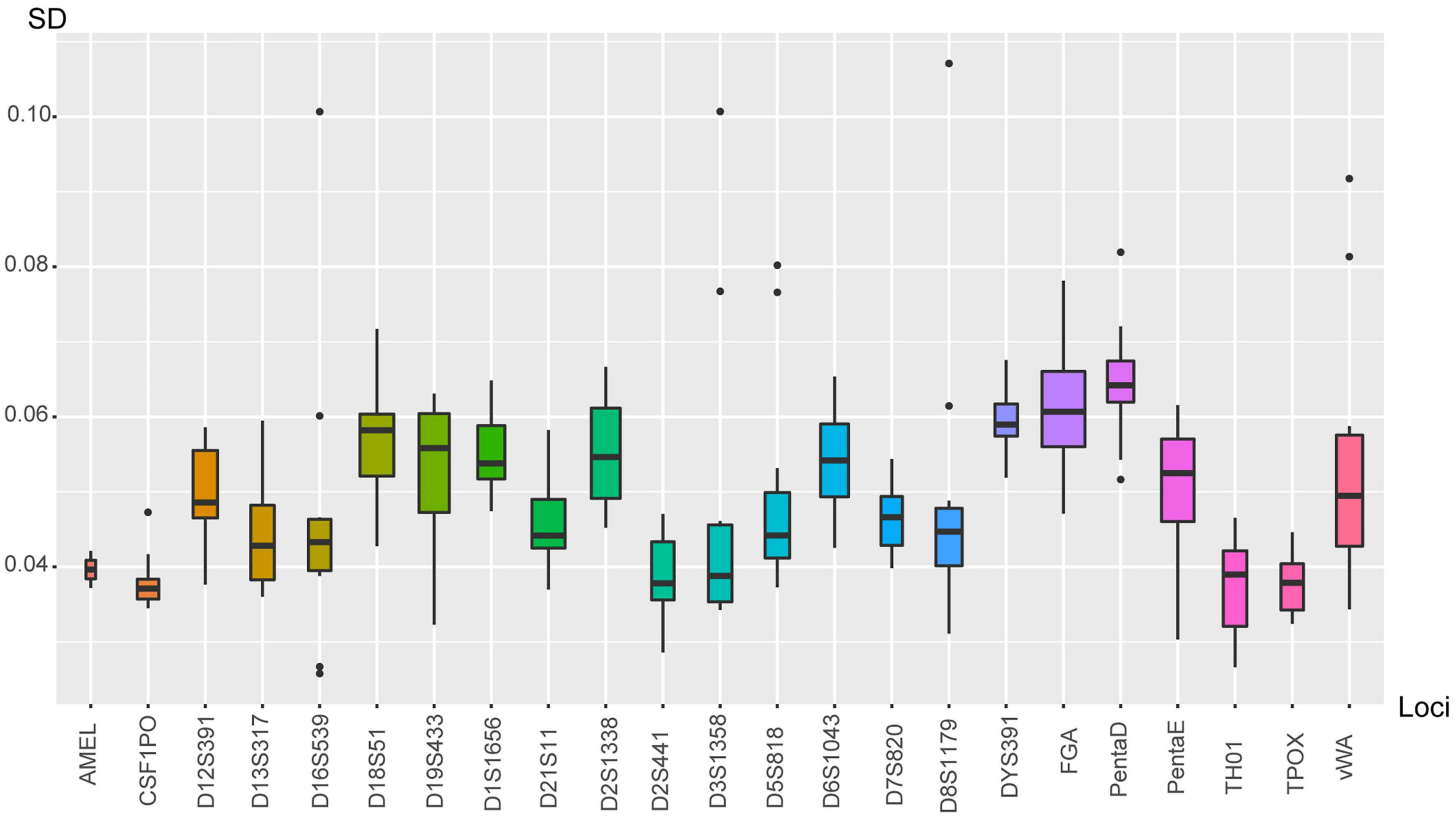
The boxplot showed the SD of allelic ladder sizing. Each box corresponded to a certain locus. Loci were listed at *x*-axis; SD values were defined as *y*-axis.

Twenty-four allelic ladders and 23 human genomic DNA extracted from 23 different samples were used to calculate the precision of the size calling and the accuracy of allele designation. The target value for the SD of allelic ladder sizing precision was no more than 0.15. In the present study, by calculating the 24 ladders across all injections, the SDs were ranged from 0.0328 to 0.1071 (Figure 2). The largest one was obtained once allele 14 at D8S1179, followed by allele 20 at D3S1358 and allele 5 at D16S539. Furthermore, none of the alleles of 23 samples fell beyond the 0.5 bp range of the corresponding position designated by the ladder. These values ensured the accuracy of the electrophoretic typing and confirmed that base pair sizing down to 1 bp was able to be identified by using the system.

### Analytical Threshold Calculation

Analytical threshold (detection threshold) helps to determine whether a peak is reliable or not. The value depends on instrument sensitivity and instrument baseline noise (Rakay et al., 2012). In this study, the maximum peak height for drop-in peaks was 40 RFUs. The average drop-in peak height was from 7.8 (blue channel) to 10.7 RFUs (purple channel). Three formulas were applied to calculate the analytical threshold (Table 1), and the maximum values obtained by each method were 26.9, 64.7, and 74 RFUs, respectively. By considering that more drop-in peaks would be detected if the analytical threshold was set too low, and a decrease of the full allele profile would be generated when it was set too high, we consider 80 RFUs would be the appropriate analytical threshold.

**Table 1:** Analytical threshold results obtained from 24 negative controls.

Parameters (RFUs)	Blue	Green	Yellow	Red	Purple
*Y*_max_	35	35	38	40	38
*Y*_min_	1	1	1	1	1
Average peak height	7.8	10.2	8.1	8.3	10.7
Average peak height + 3SD	18.9	21.3	18.3	19.1	26.9
Average peak height + 10SD	44.8	47.2	42.1	44.3	64.7
2*(*Y*_max_–*Y*_min_)	68	68	74	78	74

SD, standard deviations; *Y*_max_, highest peak height; *Y*_min_, lowest peak height.

### Sensitivity Studies and Stochastic Threshold Calculation

**Figure 3:**
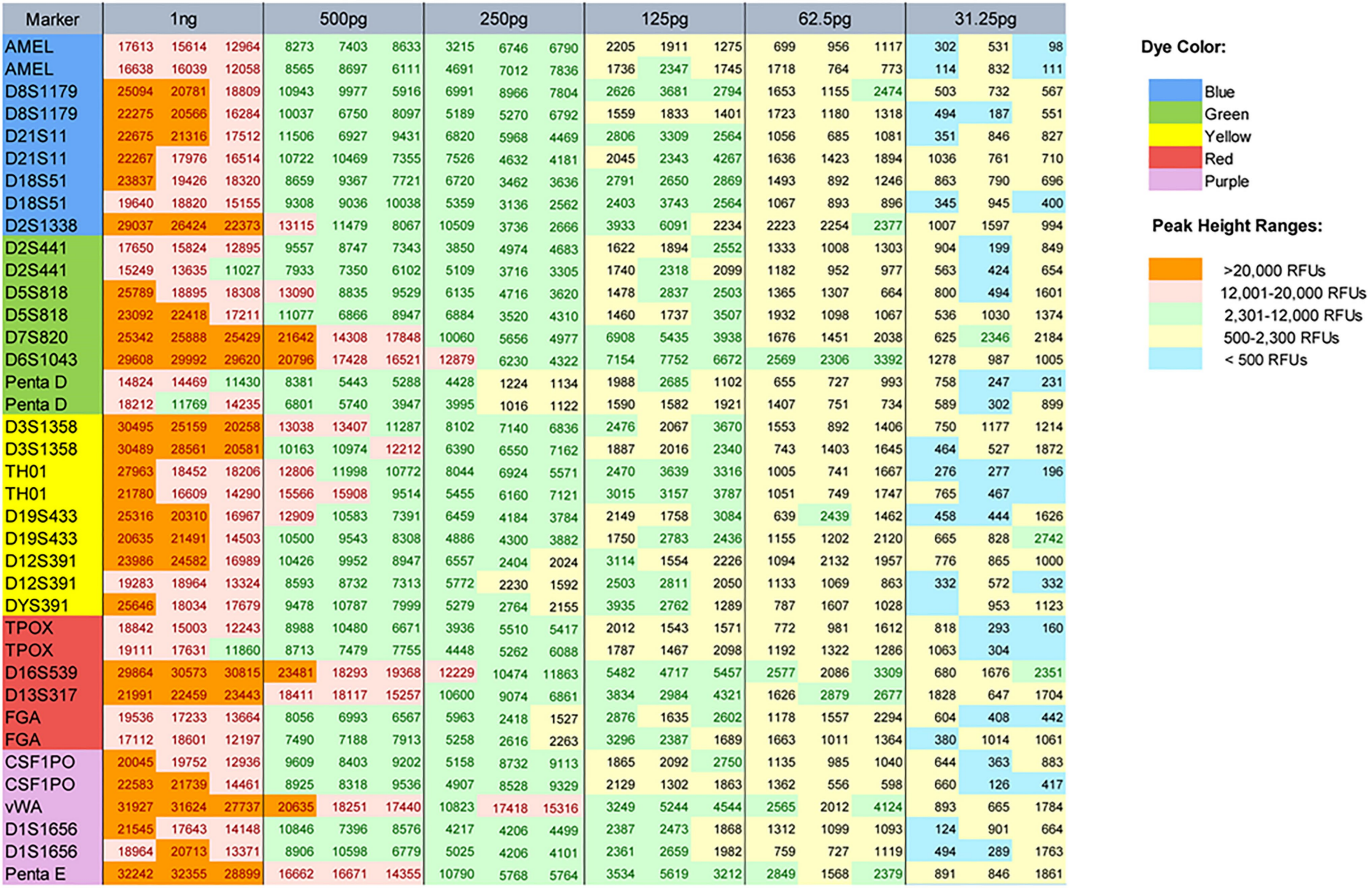
Peak heights for male control DNA 9948 when the following template amounts were amplified: 1 ng, and 500, 250, 125, 62.5, and 31.25 pg. The peak height ranges were color-coded as follows: <500 RFUs (blue), 500–2,300 RFUs (yellow), 2,301–12,000 RFUs (green), 12,001–20,000 RFUs (pink), and >20,000 RFUs (orange). The dye color of each locus and color indicating each peak height range were shown as two legends on the right of the figure.

In the sensitivity studies, by typing 1,000, 500, 250, 125, 62.5, and 31.25 pg male control DNA 9948, the allelic peak height and heterozygote balance were calculated to find the ideal range of input DNA. Full profiles were obtained from 62.5 to 1,000 pg with the threshold of 80 RFUs. The signal reduced as the template amount decreased. Fluorescent saturation and pull-up peaks appeared when the quantity of input DNA was 1 ng. Allele dropouts (alleles 9.3 of TH01 and 9 of TPOX) were observed at 31.25 pg (Figure 3). By analyzing the typing results, the ideal range of the total input template was from 125 to 500 pg. In this range, the heterozygous PHR at most circumstances was above 0.70 (the minimum PHR was 0.4980 at 125 pg).

The stochastic effects have two forms: one form is the considerable imbalance (i.e., PHR < 60%) in two alleles of a heterozygous locus, the other is allele dropout (Gill et al., 2009). The typing results of the study showed that the imbalance between peak heights increased as DNA quantity decreased (Table 2), and false homozygotes appeared when DNA quantity was reduced to 31.25 pg. Since low-level DNA tended to be impacted by stochastic effects, if only one allele was called, it was difficult to decide whether the genotyping result was real or not. For DNA samples with non-ideal quality or quantity, applying an appropriate stochastic threshold would help to distinguish whether a locus was a true or false homozygous. The stochastic threshold (i.e., dropout threshold) was generated as the RFU value when homozygote was recognized as real with a high degree of confidence. Biological sensitivity, PCR assays, and injection parameters could influence the value. By calculating the peak height of the observed false homozygotes (196 RFUs of TH01 and 160 RFUs of TPOX) at 31.25 pg, the average peak height of the observed false homozygotes was 178 RFUs with the SD of 25.5 RFUs. Finally, the stochastic threshold was suggested to set at 260 RFUs.

**Table 2:** Stochastic effects observed in sensitivity study with varying inputs of 9948 for 2+27 cycles in triple.

9948 (pg)	Loci number of PHR < 0.7	Average PHR	Lowest PHR	Lowest Het. peak (RFUs)	Highest Het. peak (RFUs)	Lowest Hom. Peak (RFUs)	Highest Hom. Peak (RFUs)
1,000	0	0.8845	0.7715	11,027, 12,895	30,495, 30,489	17,679	32,355
500	2	0.8514	0.6766	3,947, 5,288	15,908, 11,998	7,999	23,481
250	4	0.8516	0.5878	1,016, 1,224	9,113, 9,329	2,155	12,229
125	13	0.7747	0.4980	1,102, 1,921	3,296, 2,876	1,289	7,752
62.5	19	0.7327	0.4069	598, 1,040	2,474, 1,318	787	4,124
31.25	30	0.5947	0.2510	98, 111	2,742, 1,626	665	2,351

PHR, peak height ratio; Het, heterozygous; Hom, homozygous.

Based on the above experimental data, the optimum amounts of human genomic DNA were from 125 to 500 pg. When the analytical threshold was 80 RFUs, the stochastic threshold was 260 RFUs, and the typing result was of a high degree of reliability.

### Stutter Calculations

Stutter peaks are amplification artifacts mainly caused by polymerase slippage (Bennett et al., 2019). Stutter peaks often lead to confusion when dealing with mixture samples, degradation samples, or imbalanced peaks. It is helpful to use a stutter filter to remove signals in stutter positions based on size and sequence or apply a stutter model that accommodated the stutter filter information in the data analysis (Bleka et al., 2020). A previous study has shown that stutter variants with more slippage steps (*N*+2 or *N*−2, etc.) tended to yield lower amounts and lower RFUs (Li et al., 2020), so we only focused on one-step slippage by-products in the threshold calculation. In the present study, by analyzing 254 sample profiles, the alleles yielded 4,552 forward *N*+1 stutters and 8,073 backward *N*−1 stutters. D2S1338 displayed the highest mean value of forward *N*+1 stutters (0.0661) and D18S51 displayed the highest mean value of backward *N*−1 stutters (0.1165). The stutter filter threshold for each locus was determined by the mean value and the corresponding SD value. For *N*+1 stutter variants, the threshold values were in the range of 0.0512–0.2209 except for locus D18S51, which yielded no onward stutters, while for *N*−1 stutters, they were in the 0.0596–0.2253 range, and all loci displayed the corresponding stutters (Table 3).

**Table 3:** Observed stutter ratios, SDs, and stutter filter thresholds in RFUs determined for 254 samples.

Loci	*N*+1 stutters						*N*−1 stutters					
	No.	Max (%)	Min (%)	Mean (%)	SD (%)	Filter threshold (%)	No.	Max (%)	Min (%)	Mean (%)	SD (%)	Filter threshold (%)
D8S1179	238	13.92	0.28	3.05	3.79	14.42	406	15.98	4.50	9.21	1.71	14.35
D21S11	289	16.58	0.38	2.08	3.08	11.32	389	18.44	4.35	10.21	1.64	15.13
D18S51	0	0.00	0.00	0.00	0.00	0.00	158	27.31	4.58	11.65	3.63	22.53
D2S1338	54	16.05	0.45	6.61	5.16	22.09	417	20.45	6.30	10.96	1.97	16.88
D2S441	342	12.62	0.13	1.65	2.39	8.81	366	13.67	2.43	6.58	2.12	12.94
D5S818	333	12.49	0.25	2.56	3.11	11.90	358	14.16	2.74	7.70	1.87	13.31
D7S820	329	13.86	0.14	1.50	2.51	9.01	352	12.23	2.55	6.47	1.94	12.28
D6S1043	410	12.69	0.23	1.59	2.19	8.15	424	13.95	4.57	8.32	1.71	13.45
Penta D	227	10.55	0.07	1.22	1.30	5.12	388	17.80	0.46	2.20	1.25	5.96
D3S1358	229	16.35	0.20	3.41	4.72	17.57	334	16.76	5.23	10.30	1.58	15.03
TH01	96	6.90	0.21	3.14	1.65	8.09	359	7.91	0.89	3.27	1.15	6.73
D19S433	46	10.91	0.25	4.69	3.77	16.02	374	15.08	3.80	7.58	1.78	12.93
D12S391	104	16.32	0.34	5.83	5.18	21.37	397	18.49	4.92	10.95	2.51	18.48
DYS391	29	28.62	0.18	1.97	5.23	17.66	249	15.62	5.10	7.15	1.16	10.63
TPOX	77	8.06	0.23	1.75	1.97	7.66	367	15.37	1.53	3.70	1.51	8.24
D16S539	299	11.51	0.21	2.50	3.14	11.91	379	25.77	3.11	7.21	2.42	14.47
D13S317	266	9.22	0.20	1.78	2.28	8.63	389	10.52	1.50	4.96	2.27	11.75
FGA	227	13.93	0.27	3.60	4.05	15.76	423	14.72	1.94	8.53	2.09	14.80
CSF1PO	274	13.68	0.27	2.54	3.16	12.02	333	16.72	2.82	7.76	1.86	13.35
vWA	195	13.14	0.19	3.06	3.69	14.14	390	16.40	1.30	7.36	3.92	19.11
D1S1656	267	16.19	0.29	2.82	4.22	15.48	408	16.92	4.41	10.62	2.30	17.51
Penta E	221	9.27	0.25	1.59	1.83	7.08	413	16.45	0.22	5.65	2.03	11.75

No., number of the observed stutters; Max, the maximum stutter ratio of the corresponding locus; Min, the minimum stutter ratio of the corresponding locus; Mean, the mean value of the corresponding stutter ratio; SD, the standard deviation of each locus.

### Species Specificity

As the lyophilized SureID®S6 system was built based on a former SureID system with the liquid amplification reagent, the cross-species validation results of the SureID system could help us to get acquainted with the species specificity of the SureID®S6 system. The cross-species validation (Singh et al., 2019) of the SureID system in Chimpanzee showed that all the revealed loci were polymorphic and the Amelogenin gene yielded identifiable banding patterns between male and female Chimpanzees. In the present study, we only paid attention to the possible cross-reactivity of the SureID®S6 system against human-associated non-primate animals. Negative results were detectable for all of the 12 kinds of non-human samples with thresholds of 80 RFUs. The results demonstrated that the SureID®S6 system showed qualified specificity against these species (Supplementary Figure S10).

### Stability Studies

The study aims to examine the effect of five common PCR inhibitors (Elwick et al., 2018; Sidstedt et al., 2019) with the newly established solid-phase PCR reagent, and the results were compared with two widely used commercial kits: PowerPlex®21system (PP21) and VeriFiler™ Plus Amplification kit (VFP). The three kits all obtained full profiles at 200 μmol/L of hematin, 100 ng/μl of humic acid, 4 mM of indigotin, and 800 mM of tannic acid. The large amplicons gradually dropped out as the inhibitor concentration increased. Table 4 showed the corresponding inhibitor concentrations when profiles were full-called, half-called, or non-called. By comparison, the S6 system and the PP21 had more advantages than the VFP kit when tested hematin, indigotin, and tannic acid. For calcium ion, only the S6 system obtained full profiles at 3 mM, while PP21 and VFP kits only called 50% alleles at the same concentration. For humic acid, the VFP and PP21 showed excellent resistance to the inhibitor, whereas the S6 system was relatively weak. When the humic acid was 250 ng/μl, alleles of the VFP kit were 100% called, while less than half of the alleles were called by the S6 system (Supplementary Figure S11).

**Table 4:** The table showed the corresponding concentrations of the inhibitors added per reaction when alleles were full called, half called, or non-allele called.

System	SureID®S6			PP21			VFP		
Call rates	100%	50%	0%	100%	50%	0%	100%	50%	0%
Hematin (μmol/L)	500	800	1,500	500	>800	1,500	200	500	800
Humic acid (ng/μl)	100	>150	>250	300	350	>350	300	350	>350
Indigotin (mM)	4	7	>15	4	>7	>15	4	<7	15
Tannic acid (mM)	800	1,500	>2,500	800	1,500	2,500	800	1,500	2,500
Calcium ion (mM)	3	10	12	/	3	10	/	3	>12

Three systems (SureID®S6, PowerPlex®21system, and VeriFiler Plus kit) were applied in this study.

To evaluate the applicability of S6 to degradation samples (Ballari and Martin, 2013), genomic DNA 9948 was exposed to UV light for 0, 2.0, 4.0, and 6.0 h at room temperature, respectively. Theoretically, the significant degradation effect would increase as time goes on, and larger amplicons would be more sensitive. For samples exposed to UV light shorter than 4.0 h, they were moderate degradation samples and obtained full STR profiles. When the time extended to 6.0 h, the severely degraded DNA sample could only yield partial STR alleles (Supplementary Figure S12).

To evaluate the stability of the S6 system, PCR reagents stored at room temperature and −20°C for 26 months were amplified to observe their performance. After repeated freezing and thawing by 10 times, the profiles revealed no significant differences in typing accuracy and peak morphology (Supplementary Figure S13).

Compared with the traditional aqueous reagents, the lyophilized reagents of the S6 system showed stronger resistance against some PCR inhibitors. The results of amplification did not decrease significantly within the shelf life or repeated freezing and thawing 10 times.

### Mixture Studies

**Figure 4:**
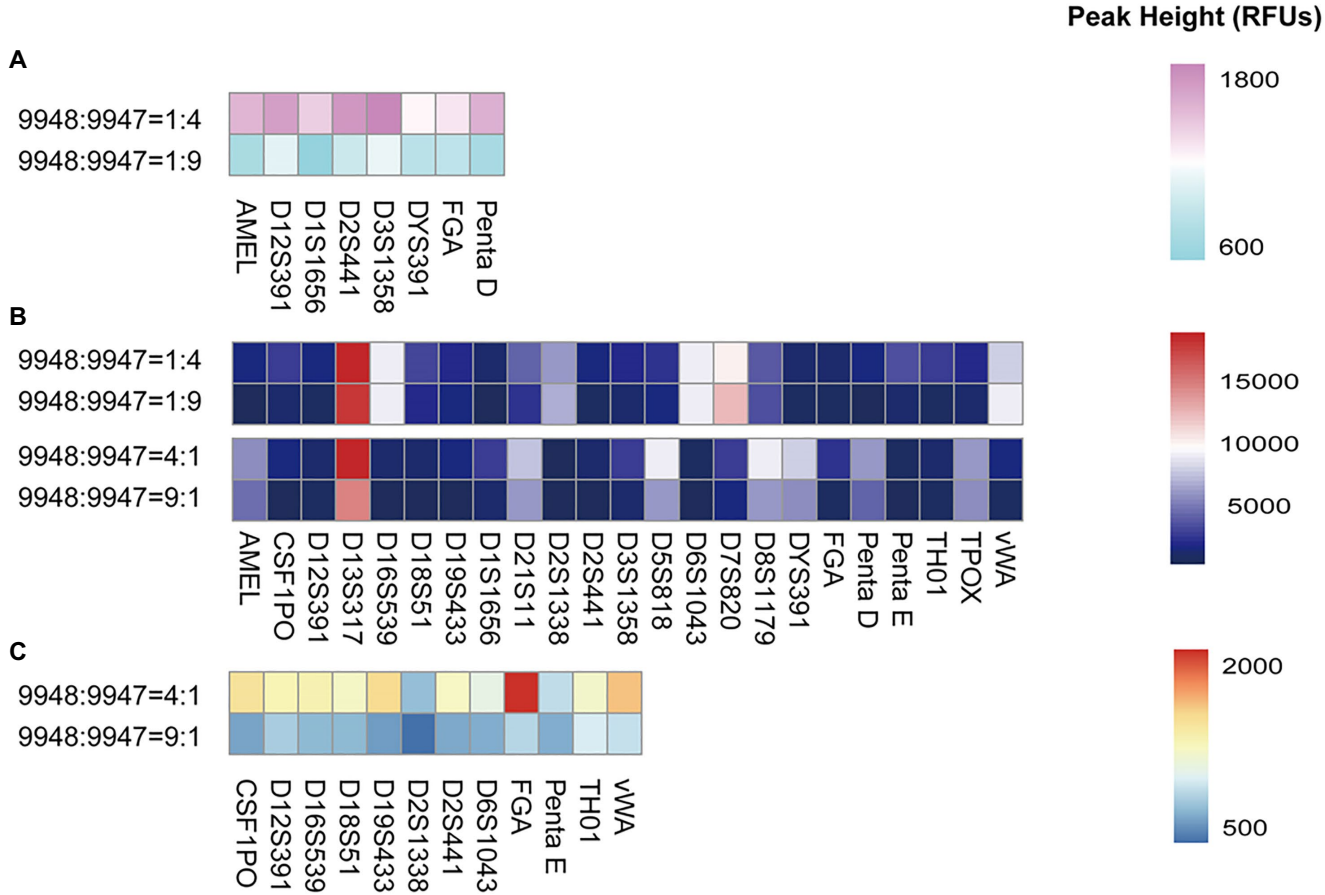
The heatmap showed the height of the lowest peak of each locus when 9948 and 9947A were mixed with the following ratios: 1:4, 4:1, 1:9, and 9:1. **(A)** peak heights under 2,000 RFUs were exhibited when 9948/9947A ratios were set at 1:4 and 1:9; **(B)** peak heights of all loci were exhibited when 9948/9947A ratios were set at 1:4, 1:9, 4:1, and 9:1; **(C)** peak heights under 2,000 RFUs were exhibited when 9948/9947A ratios were set at 4:1 and 9:1.

Mixture samples often occur in forensic casework and are relatively difficult to interpret. To explore the ability of the S6 system in mixture typing, we prepared a series of male/female mixtures with the ratios of 1:4, 4:1, 1:9, 9:1, 1:19, and 19:1. A heatmap intuitively showed the peak heights of the minor contributors (Figure 4). Full profiles of the minor contributors were obtained at the ratios of 1:1, 1:4, 4:1, 1:9, and 9:1. However, at 1:9 or 9:1, some peak heights were relatively low, only a few 100 RFUs. It was estimated that allele dropouts may occur if the DNA amount of the minor contributor was less than 100 pg. When the ratio was 19:1, the minor contributor 9947A (50 pg) hardly yielded reliable profiles for analysis. Meanwhile, at the ratio of 1:19, the minor contributor 9948 lost all loci except DYS391, which could help to distinguish the existence of the male component in the mixture.

Besides, to evaluate the male/male/female mixture, genomic DNA 007, 9948, and 9947A were mixed at the ratio of 1:1:8. The alleles of the three genomic DNA were successfully called. However, a lot of the peak heights of the male components were lower than the analytical threshold. Some of them were even lower than the stutter peaks of 9947A.

### Case Samples and Heterozygous Balance Studies

Forensic samples often come from different tissues and are found on various media. In the study, we examined 24 blood specimens, 21 buccal swabs, 20 epithelial cell samples abrasions from crime scenes, 11 semen samples, seven costal cartilage bones, one tooth, and three fragments of bones. All of them rendered full profiles with acceptable peak morphology (Supplementary Figure S14, S15). The results demonstrated that the S6 could meet the requirement to amplify various kinds of forensic samples.

### Population and Genotype Concordance Studies

A total of 500 unrelated individuals from the Zhejiang Han population participated in the population study. As the 21 autosomal STR loci of the S6 system were commonly used and repeatedly verified, there was no doubt that they were highly informative and discriminating in the Zhejiang Han population. By calculating the population data, the CPD was over 0.999999999999 and CPE was 0.999999998416. The other forensic parameters of 21 autosomal STR loci were listed in the Supplementary Table S2. No departure from Hardy-Weinberg Equilibrium was observed in these loci when the value of *p* was 0.05 (Ballari and Martin, 2013).

**Figure 5:**
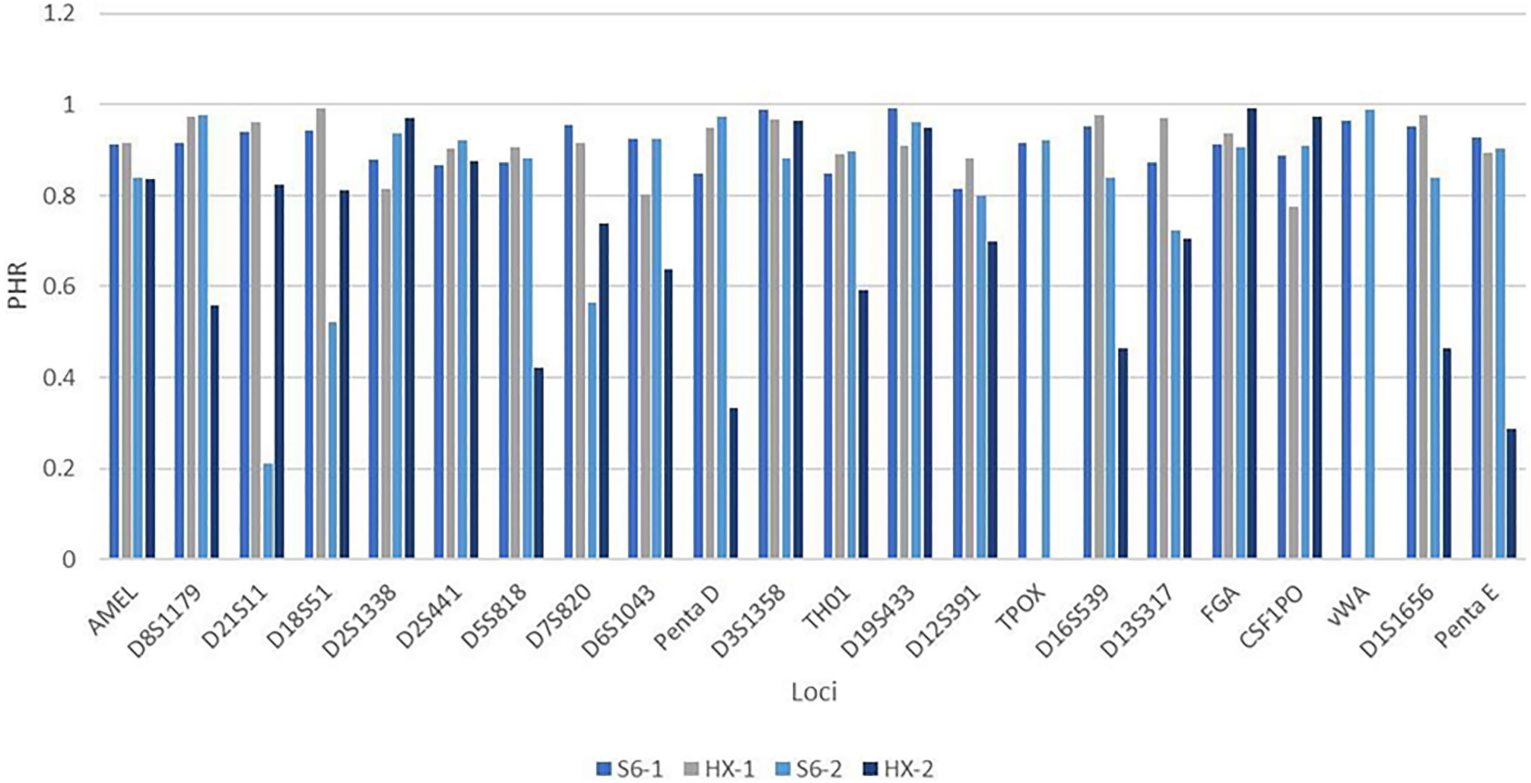
The bar plot showed the PHR values of sample 1 and sample 2 when tested by HX kit and S6 system. The *y*-axis was the PHR value and the *x*-axis was the locus. For figure legends listed on the bottom of the plot, S6-1 represented that sample 1 was amplified with S6; HX-1 represented that sample 1 was amplified with HX; S6-2 represented that sample 2 was amplified with S6; and HX-2 represented that sample 2 was amplified with HX. The height of each bar represented the PHR value of each locus.

In the genotype concordance study, the HUAXIA™ Platinum kit (Zhong et al., 2019) was employed to type the 500 individuals. As no discordance was observed by checking the profiles of the two systems, the S6 system was proved to be reliable (Supplementary Table S3). Then, two males with high heterozygosities were chosen to compare the intra-balance between the two kits. For sample 1, the average peak height of the S6 system was 6,543 RFUs, while it was 2,445 RFUs for the HUAXIA™ Platinum kit; for sample 2, the average peak heights were 8,994 and 7,090 RFUs, respectively. The bar plot (Figure 5) showed that PHR values for sample 1 were over 70%. For sample 2, three loci (D21S11, D18S51, and D7S820) of the new system fell behind 70%, while eight loci fell behind 70% (D8S1179, D5S818, D6S1043, Penta D, TH01, D16S539, D1S1656, and Penta E) for HUAXIA™ Platinum kit (Supplementary Figure S16). The results of population and concordance studies confirmed that the dry PCR reagents were accurate and reliable for individual identification and paternity testing, just like the traditional systems with liquid reagents.

## Conclusions

Dry amplification reagent avoids possible contamination in the solution preparation process, and reduces transportation costs by prolonging the storage time at a temperature above 0°C. The SureID®S6 PCR amplification system employed lyophilized dry amplification reagent is designed with shortened fragment length (<400 bp), and a pair of IQCs. The system is capable of amplifying purified DNA from caseworks and database samples on FTA™ cards in a direct amplification module with a 10 μl reaction volume. The validation studies were conducted following the SWGDAM guideline, and the results displayed this new dry system was suitable for forensic investigation. Thus, we recommend the new kit as a choice for forensic DNA testing.

## Data Availability Statement

The original contributions presented in the study are included in the article/Supplementary Material; further inquiries can be directed to the corresponding author.

## Ethics Statement

The studies involving human participants were reviewed and approved by the Ethical Committee of Xi’an Jiaotong University, China (Approval Number: 2019-1039). The participants provided their written informed consents to participate in this study.

## Author Contributions

BZ: conceptualization, project administration, and funding acquisition. JL: methodology and resources. SL: software, formal analysis, investigation, writing – original draft preparation, visualization, and validation. HH and JL: validation. HJ and HH: data curation. HJ and DS: writing – review and editing. BZ and DS: supervision. All authors contributed to the article and approved the submitted version.

## Conflict of Interest

HJ and DS were employed by the company HNingbo Health Gene Technologies Co., Ltd, Ningbo, Zhejiang Province, China.

The remaining authors declare that the research was conducted in the absence of any commercial or financial relationships that could be construed as a potential conflict of interest.

## Publisher’s Note

All claims expressed in this article are solely those of the authors and do not necessarily represent those of their affiliated organizations, or those of the publisher, the editors and the reviewers. Any product that may be evaluated in this article, or claim that may be made by its manufacturer, is not guaranteed or endorsed by the publisher.

## Footnotes

^1^ https://1ecb9588-ea6f-4feb-971a-73265dbf079c.filesusr.com/ugd/4344b0_813b241e8944497e99b9c45b163b76bd.pdf

^2^ https://www.promega.com/-/media/files/resources/validation-guides/internal-validation-of-autosomal-str-systems.pdf?la=en
