# Supplementary material for: Validation of a 6-Dye Short Tandem Repeat System: A Dry Kit With Lyophilized Amplification Reagent
Source: Front Genet. 2021 Sep 6;12:705819. doi: 10.3389/fgene.2021.705819 (PMC8451954; doi:10.3389/fgene.2021.705819)
Supplement: Supplementary file 1 [file Table_1.DOCX]

Supplementary Material

# Supplementary Figures and Tables

## Supplementary Figures (S1-S16)


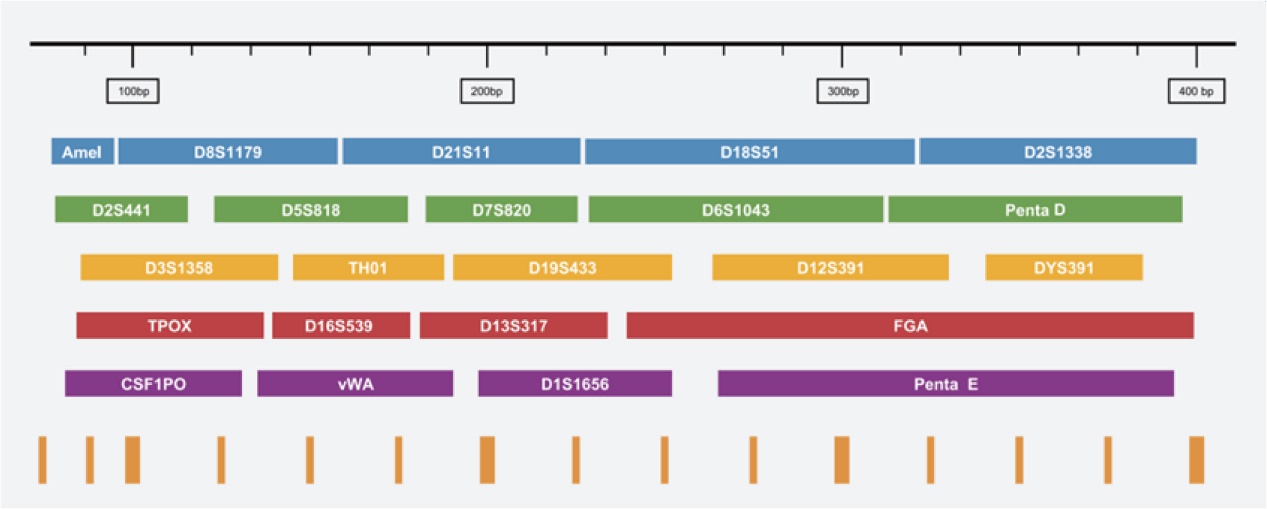


Supplemental Fig. S1. Map of loci distribution.


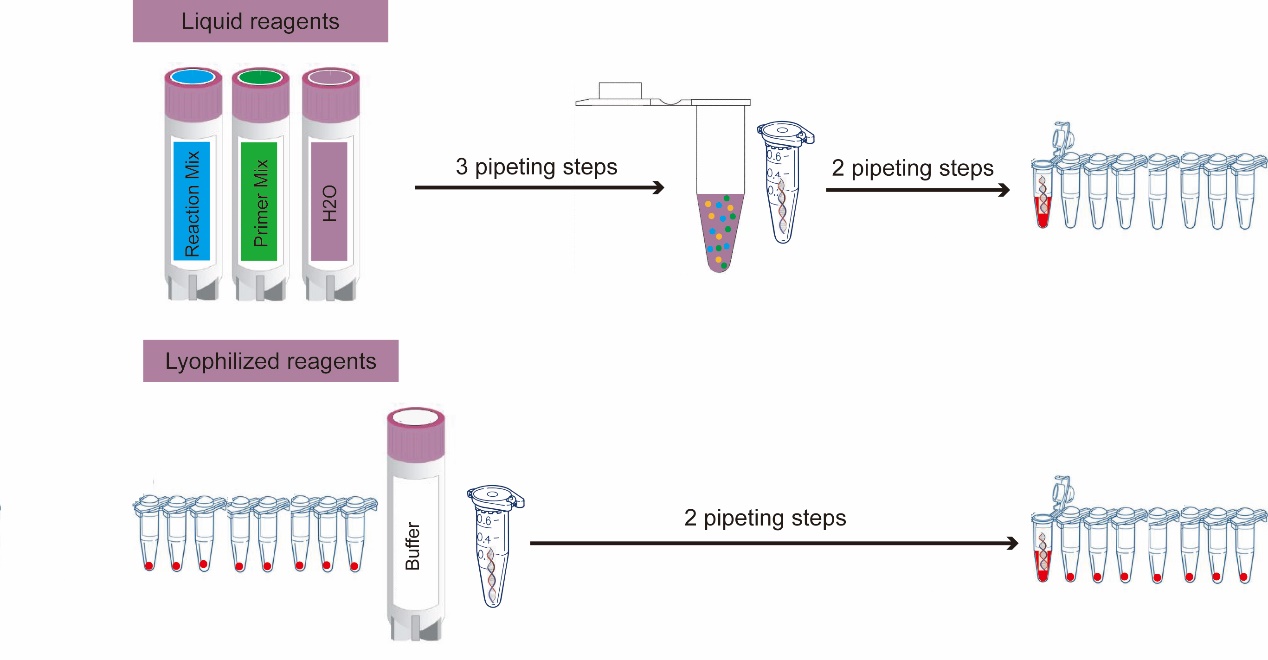


Supplemental Fig. S2. The comparison between liquid reagents and lyophilized reagents. With the ready-to-use freeze-dried PCR mixtures, the process of preparing premixture can be omitted, which can simplify the operation procedures to a maximum extent.


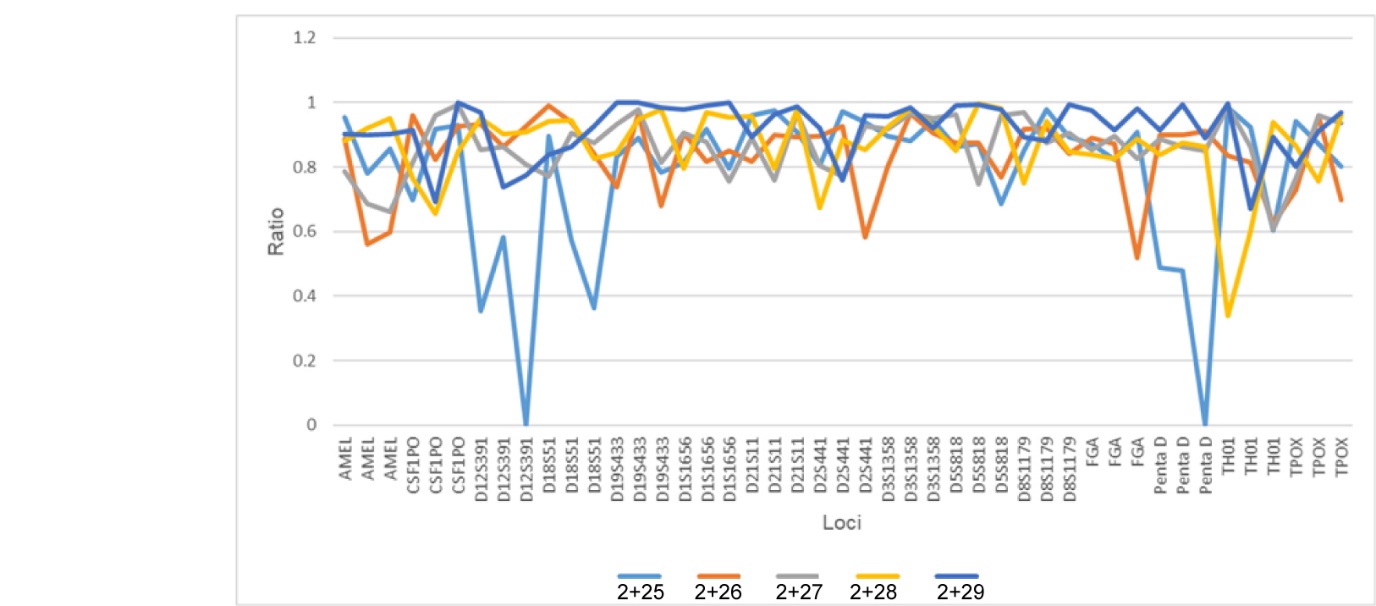


Supplemental Fig. S3. Cycle number tests for 0.5 ng 9948 DNA.


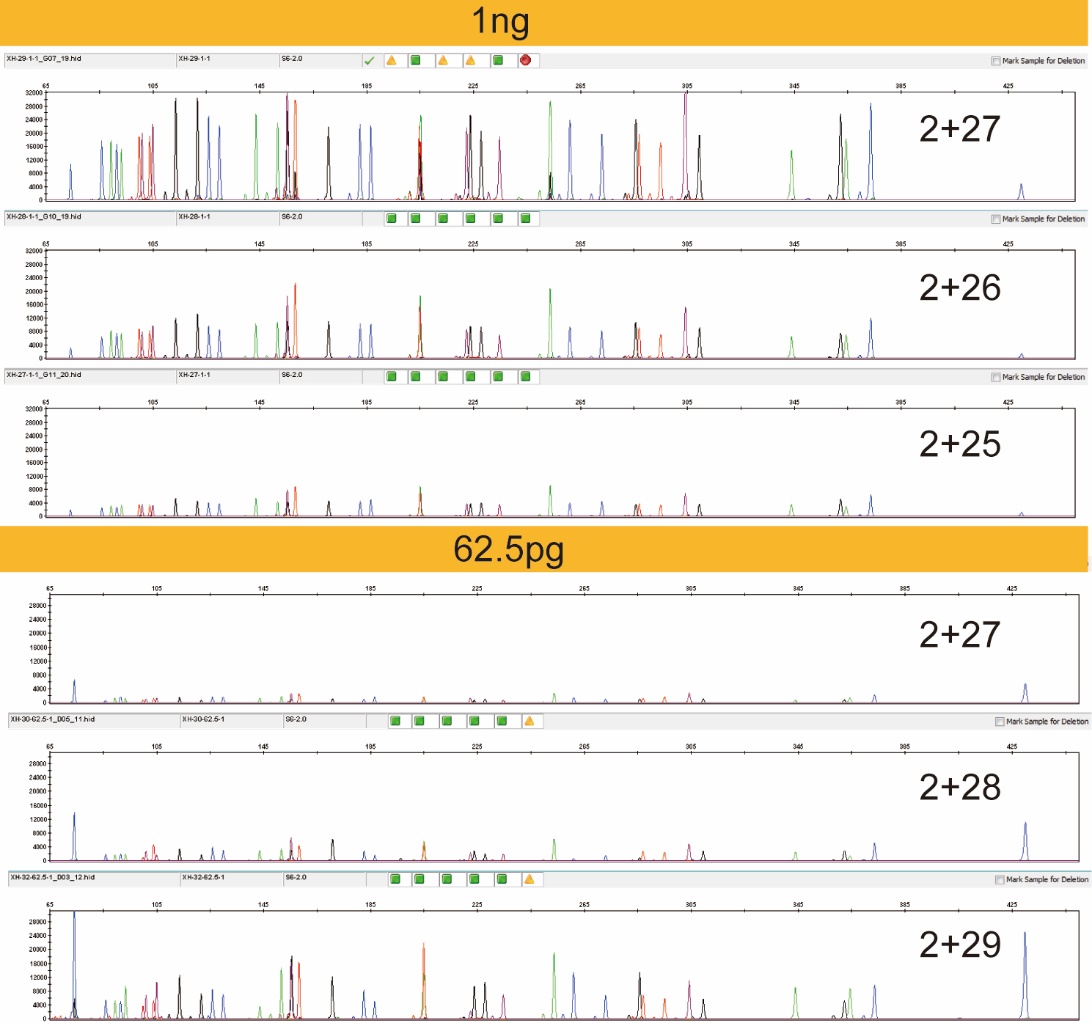


Supplemental Fig. S4. Cycle number test for 1ng and 0.0625ng 9948 DNA.


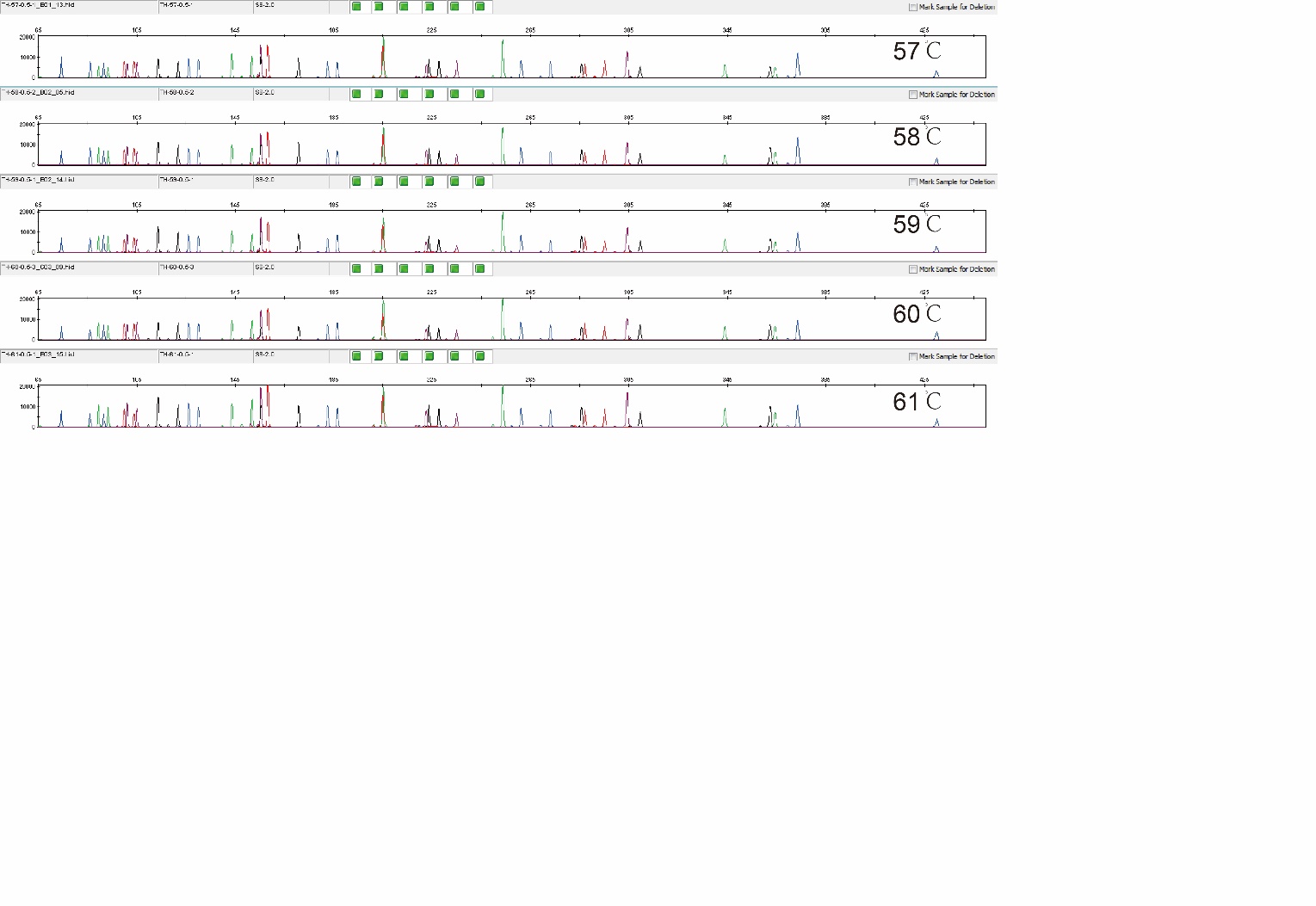


Supplemental Fig. S5. Annealing temperature study for 0.5 ng of 9948 DNA.


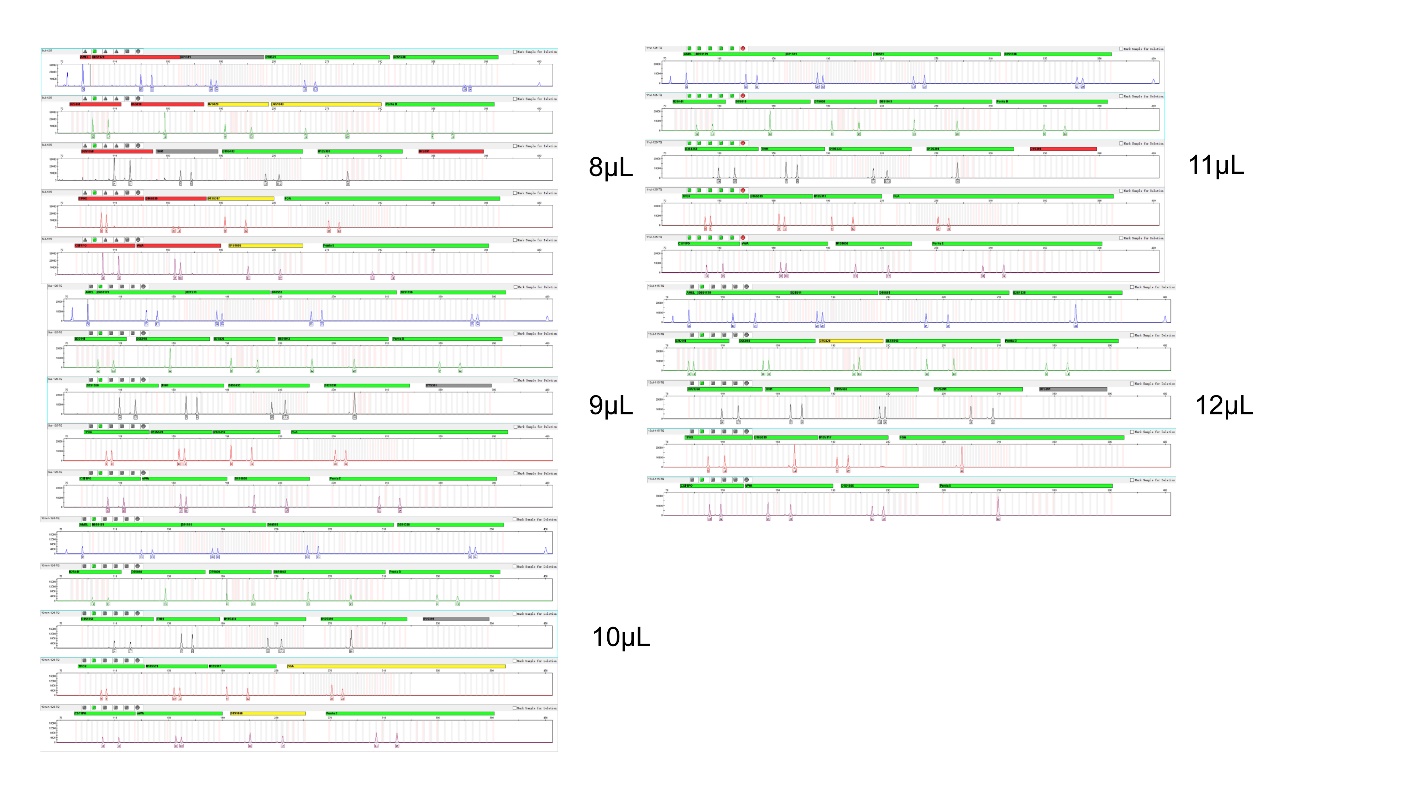


Supplemental Fig. S6. The volume study tested the reaction volume of 8 μL, 9 μL, 10 μL (recommended), 11 μL and 12 μL with extracted DNA. When the volume was 8 μL, partial profile showed unsatisfying heterozygous balance. The intra-locus balance becomes better as the volume increases.


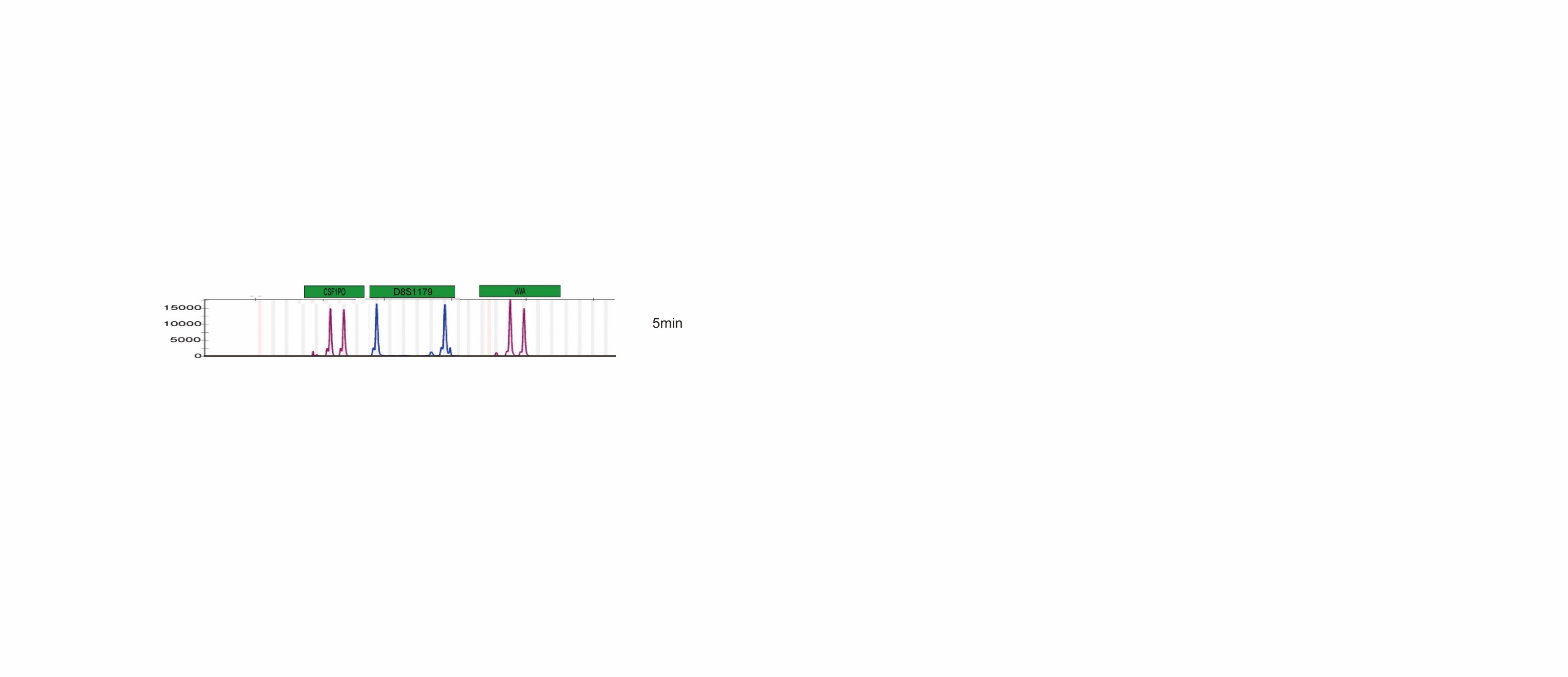


Supplemental Fig. S7. Final extension time tests for three extracted DNA. Normal peak morphology was obtained for the 5 min, 10 min, 15 min and 30 min hold time, but CSF1PO, D8S1179 and vWA had small shoulder peaks for the 5-min-hold.


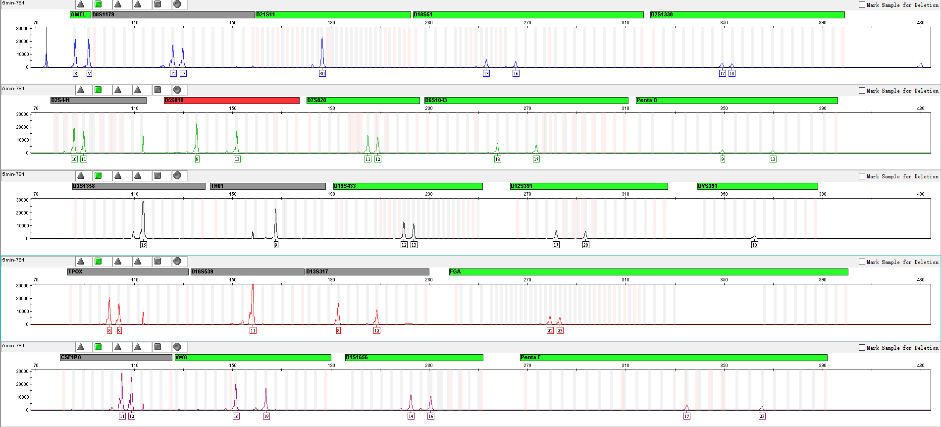
 **5min**


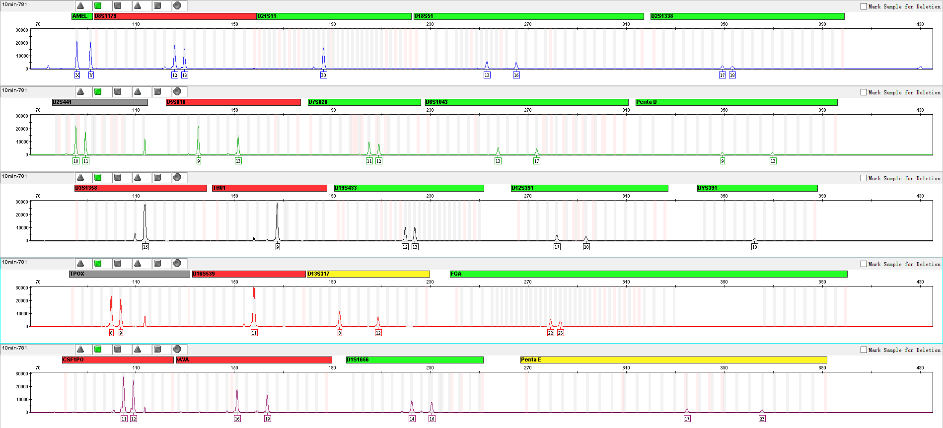
 **10min**


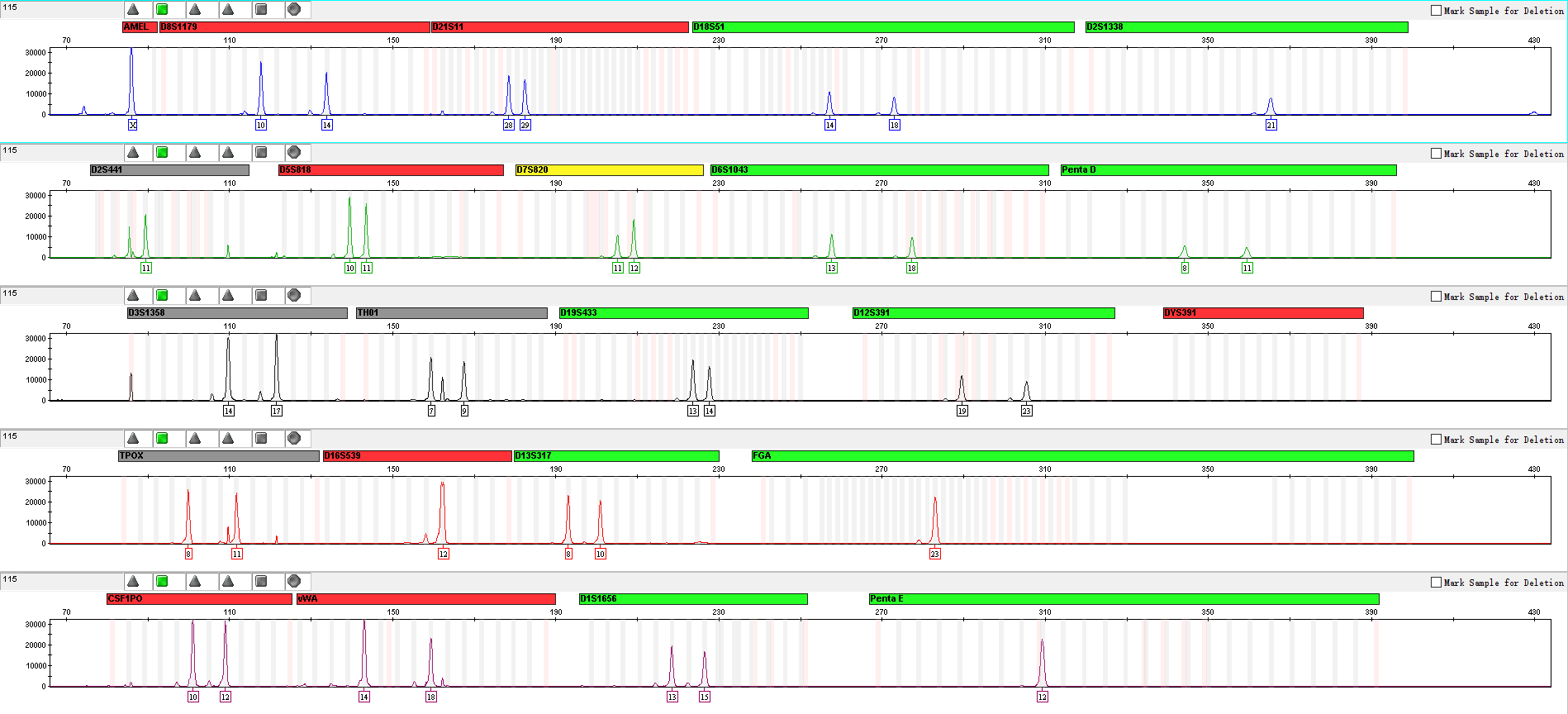
 **15min**

Supplemental Fig. S8. Extension time tests for three blood FTA^®^ cards, nine loci showed minus A peaks for the 5-min-hold, 4 loci (D8S1179, TPOX, CSF1PO and vWA) showed minus A peaks for the 10-min-hold. Complete terminal nucleotide addition was obtained for 15 min.


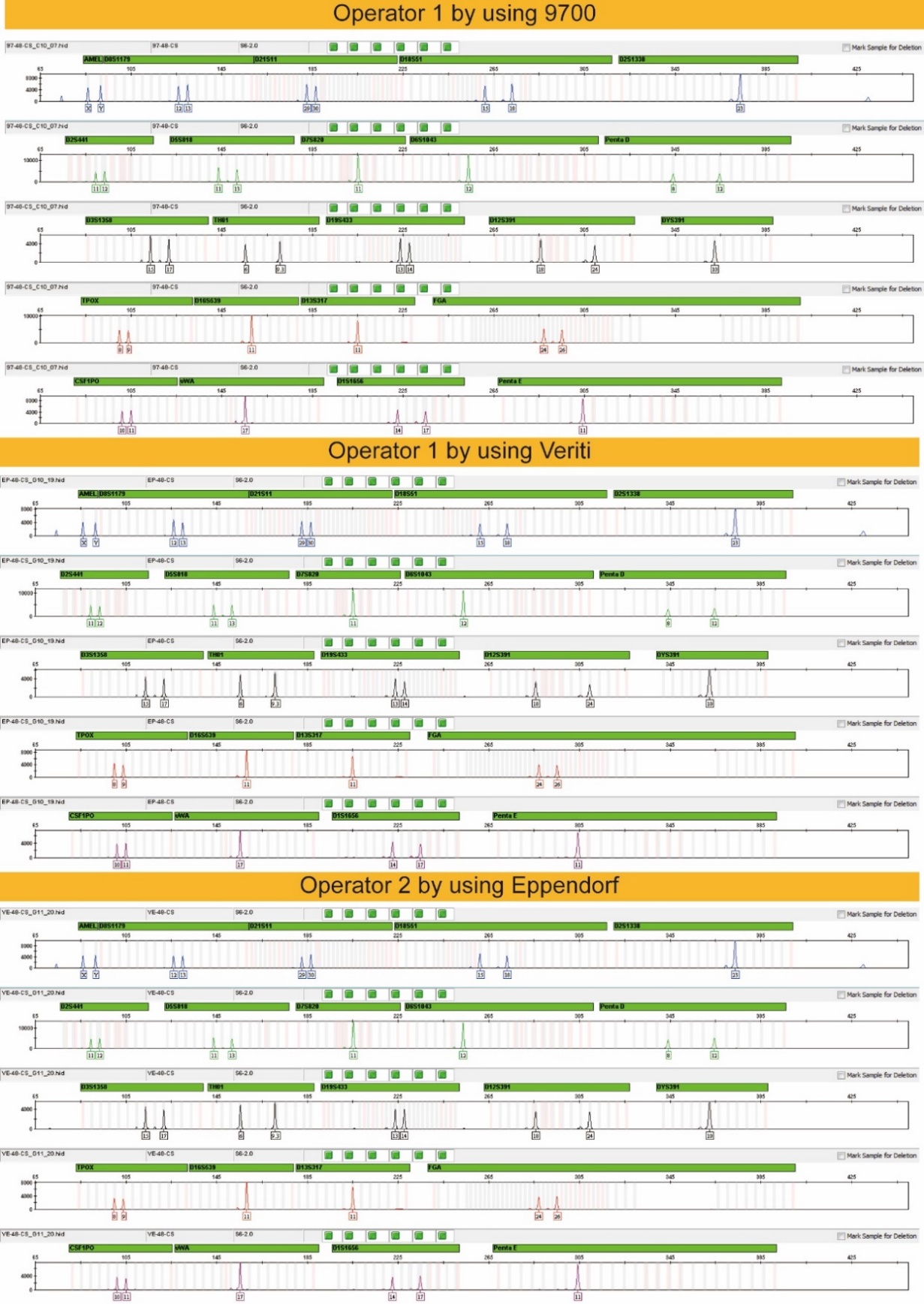


Supplemental Fig. S9. In the reproducibility study, the expected genotypes obtained by using 3 different PCR instruments were concordant across 9948 and two male DNA samples and across two different operators.


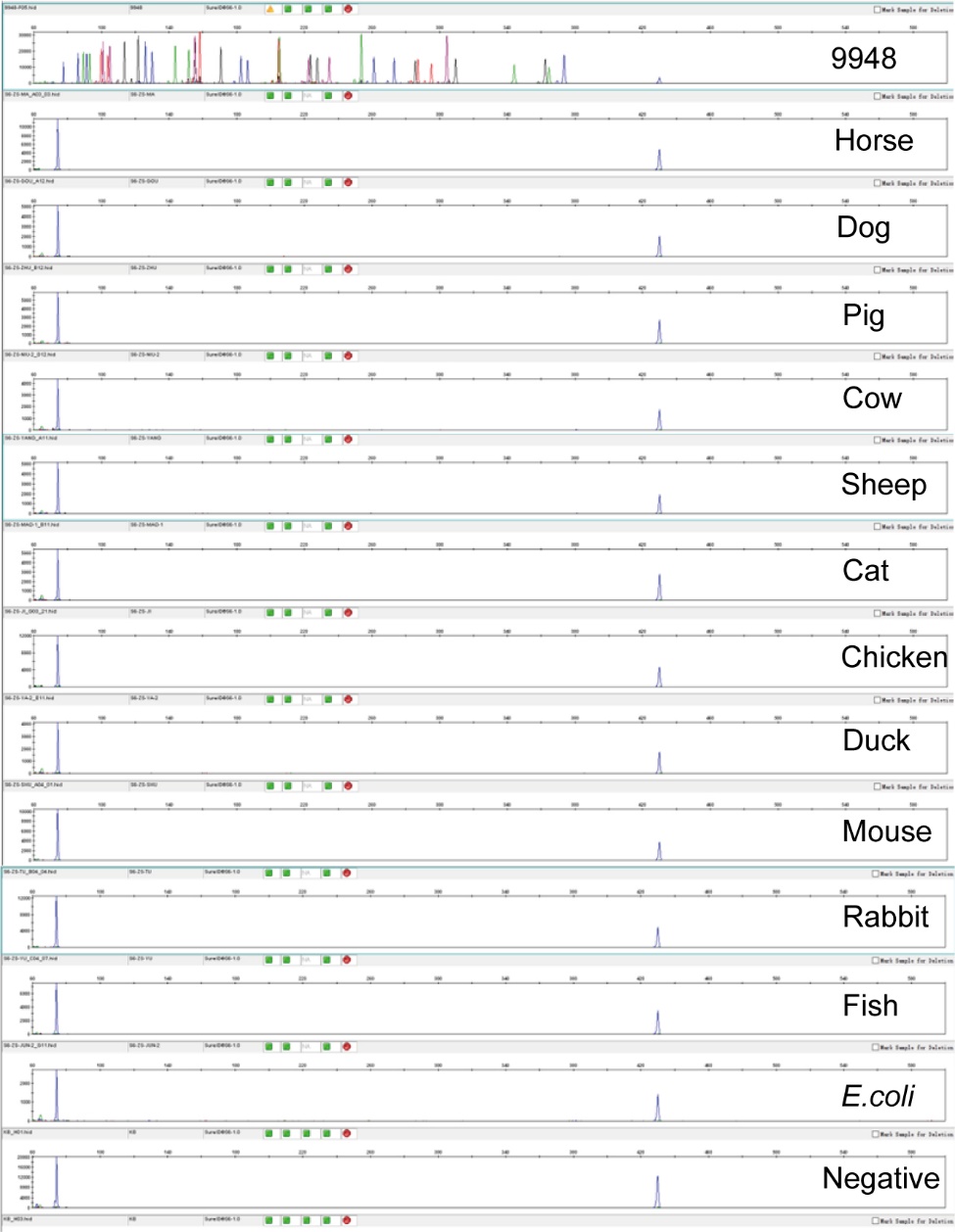


Supplemental Fig. S10. The profiles of twelve kinds of non-human genomic DNA samples which were tested for cross-reactivity with the SureID^®^ S6 System.


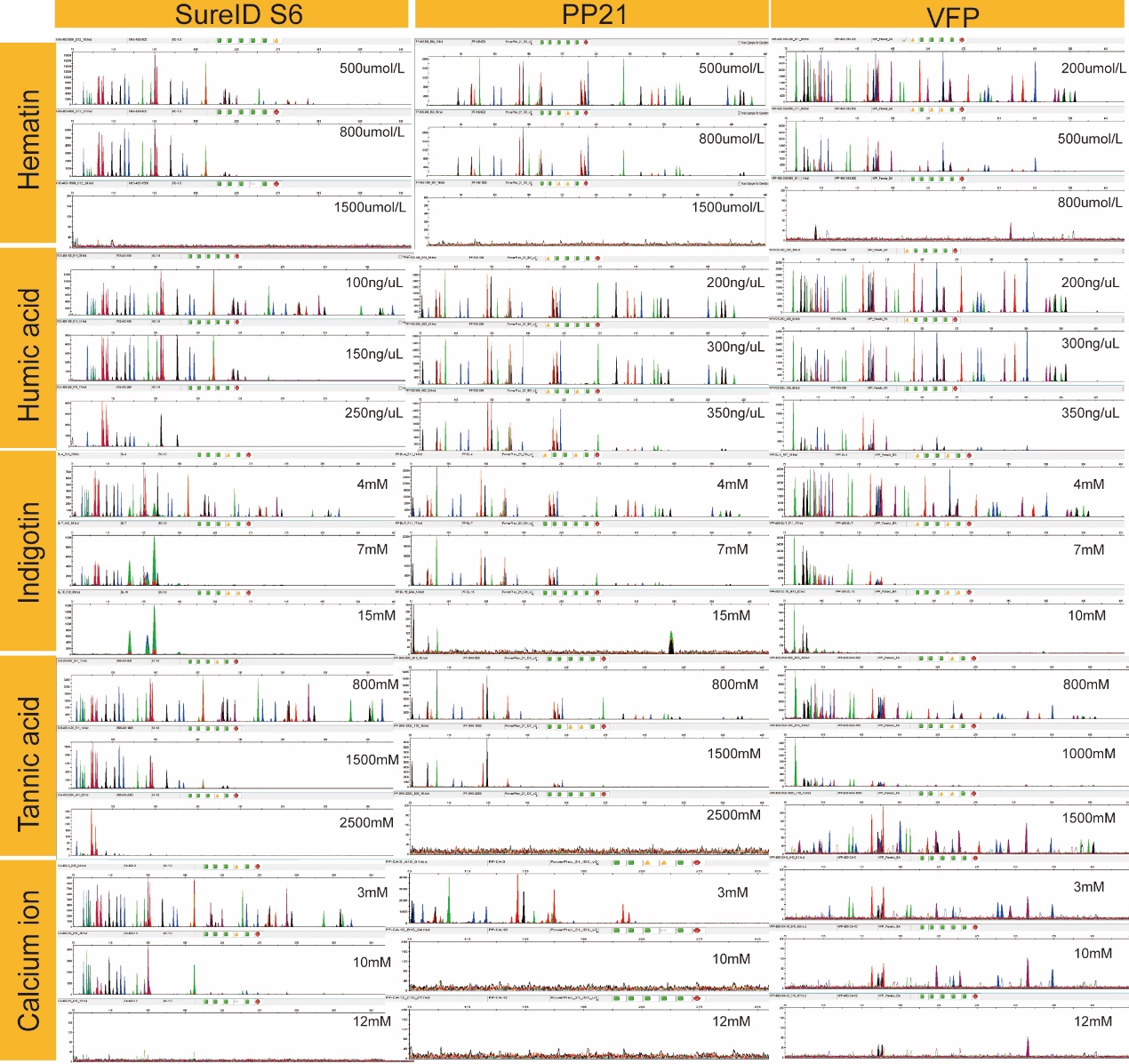


Supplemental Fig. S11. Comparison of the inhibition among SureID® S6 system, PowerPlex21system (Promega) and VeriFiler Plus kit (Thermo Fisher)


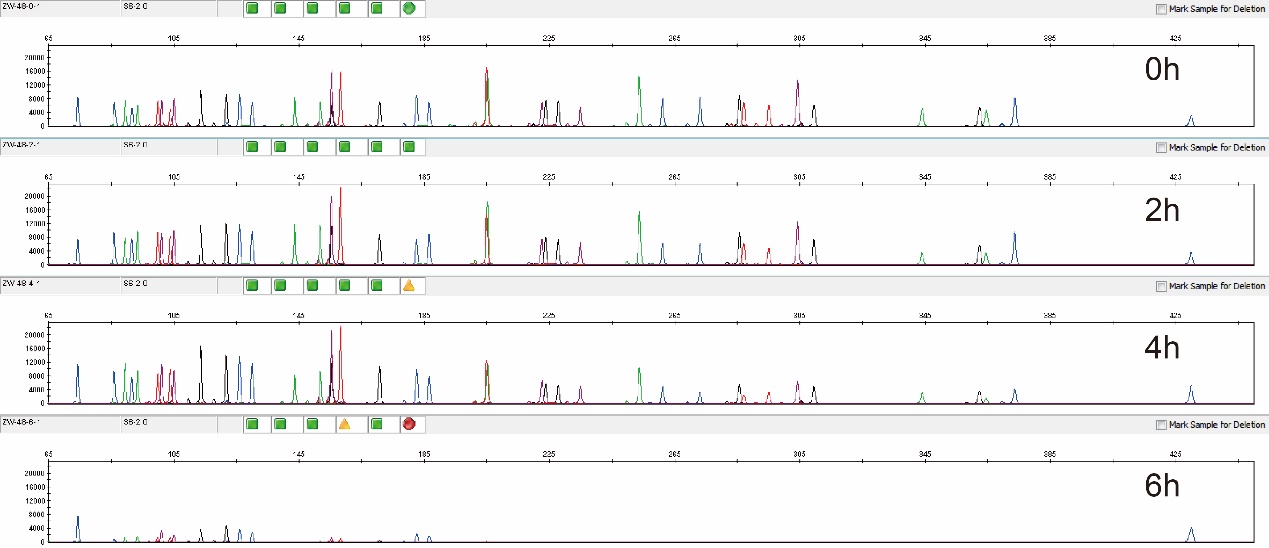


Supplemental Fig. S12. The profile of 9948 when it was exposed to UV light for 0 h, 2 h, 4 h and 6 h at room temperature.


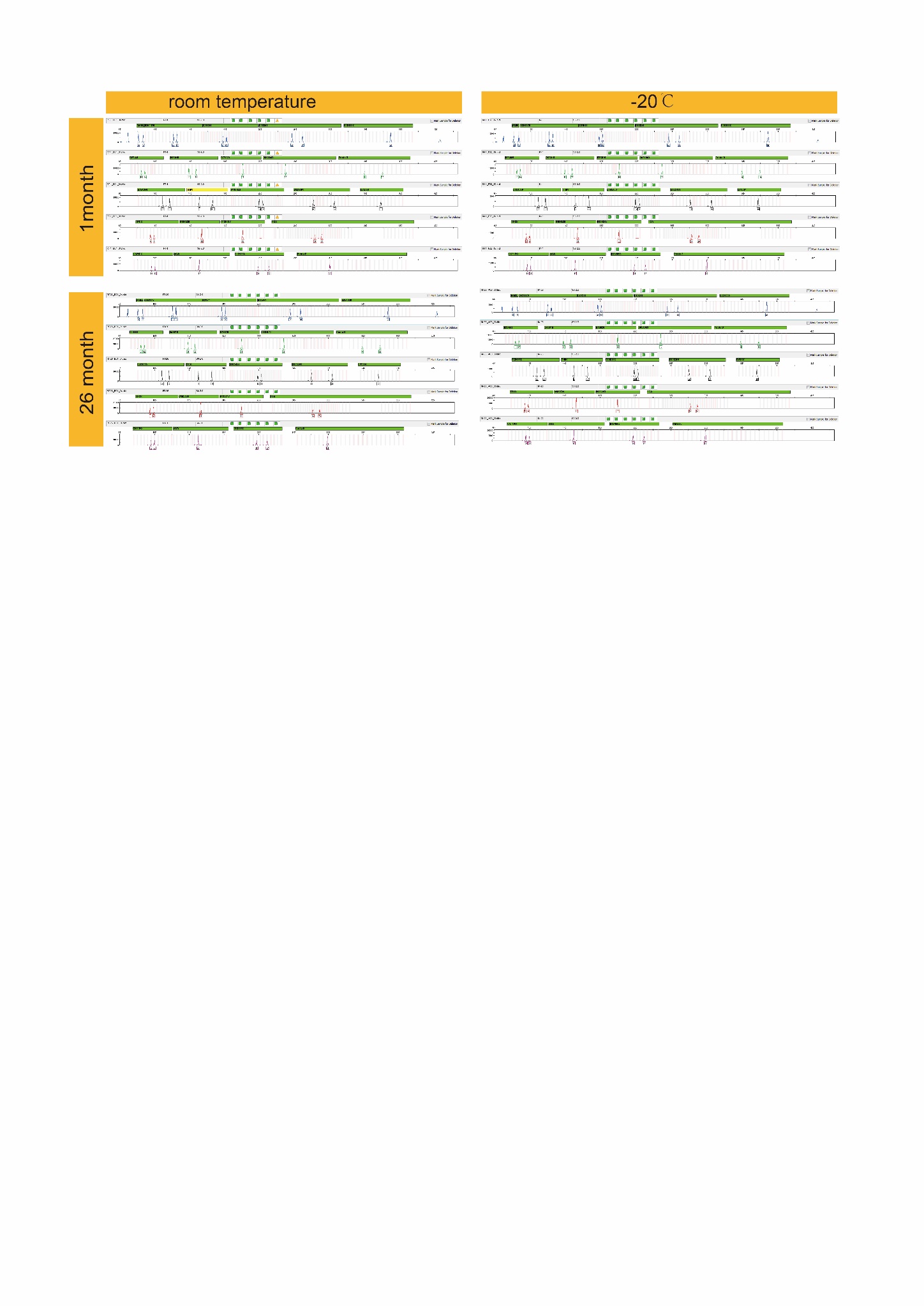


Supplemental Fig. S13. To evaluate the stability of the kit, PCR reagent stored at room temperature and -20℃ for 26 months was tested and there is no significant difference in the effect of 9948 typing.

1. Blood FTA^®^ card:


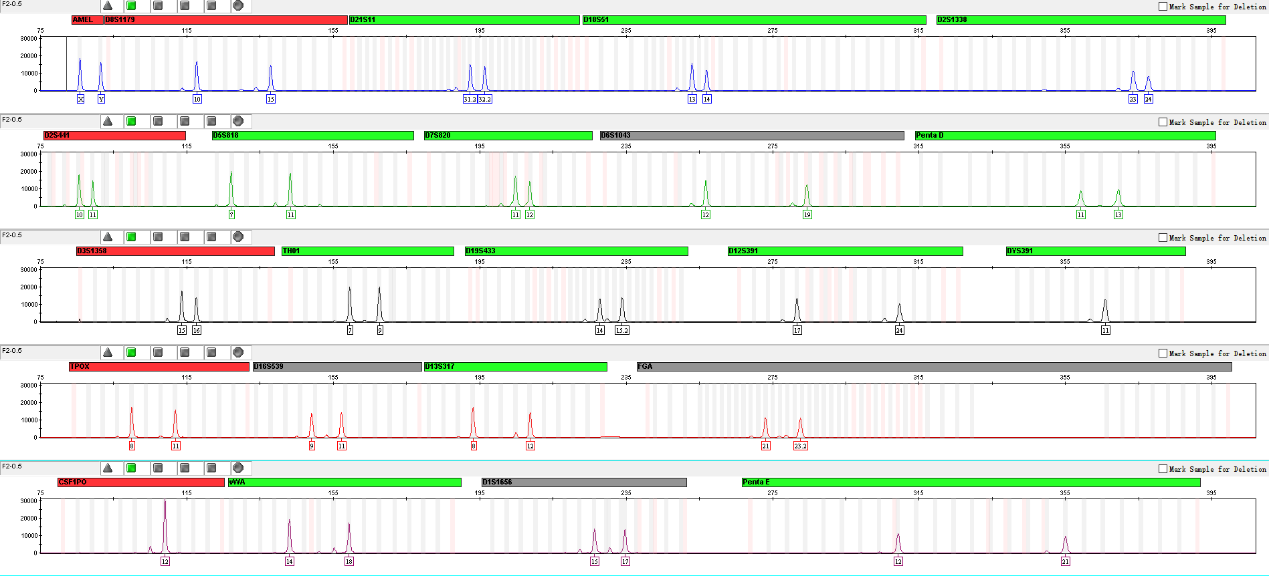


1. Buccal-indicating FTA^®^ card:


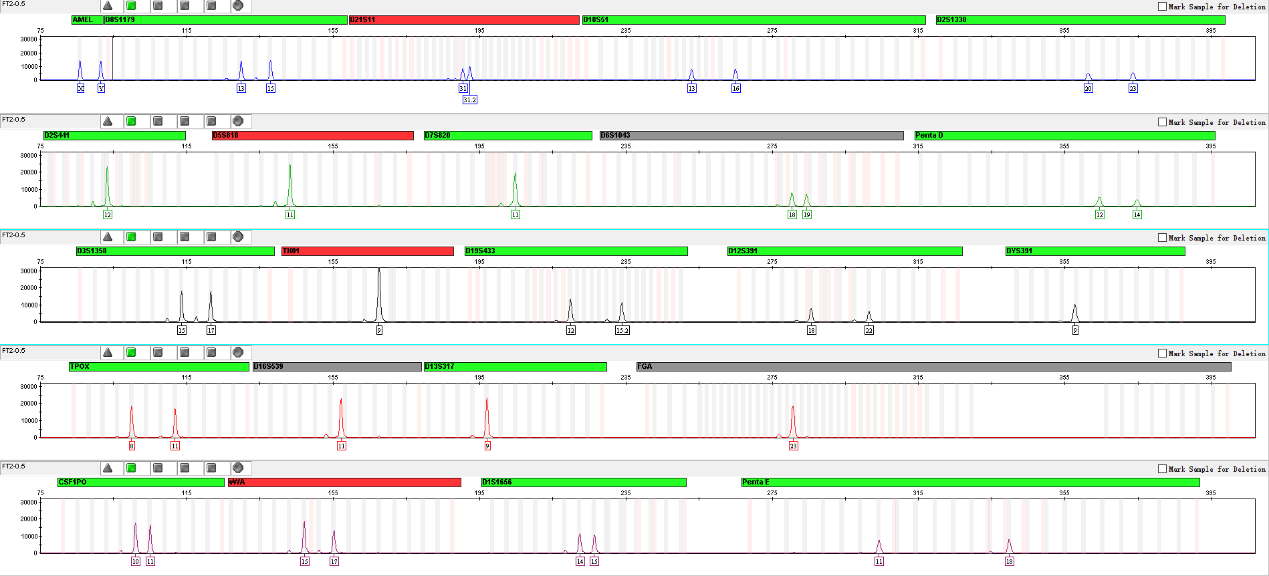


1. Filter paper:


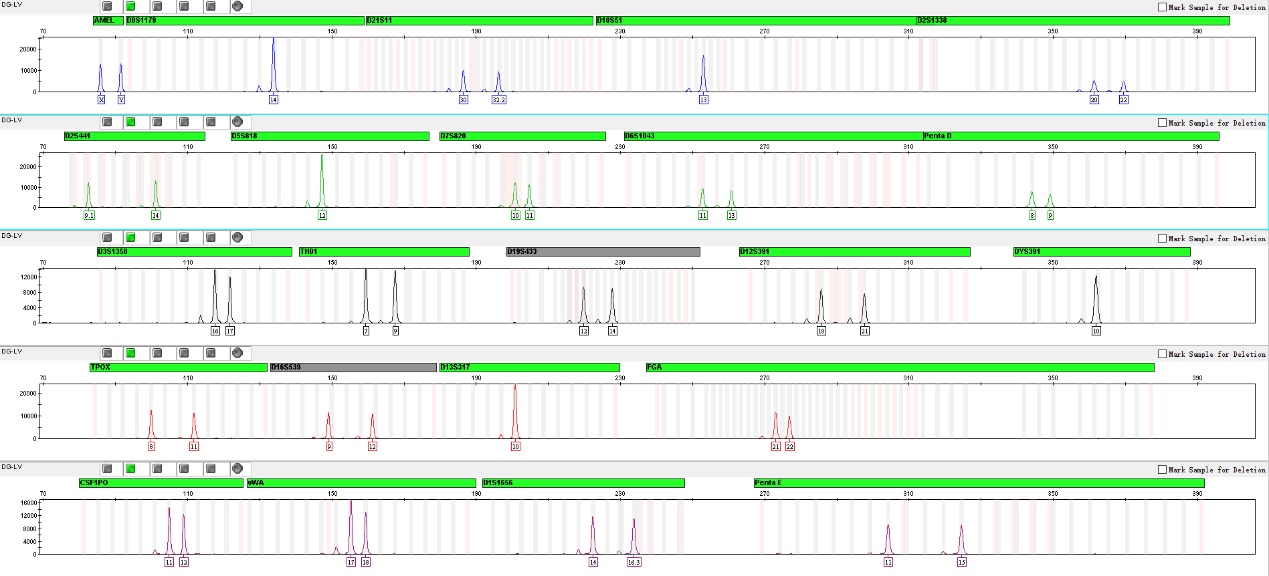


1. Buccal swab:


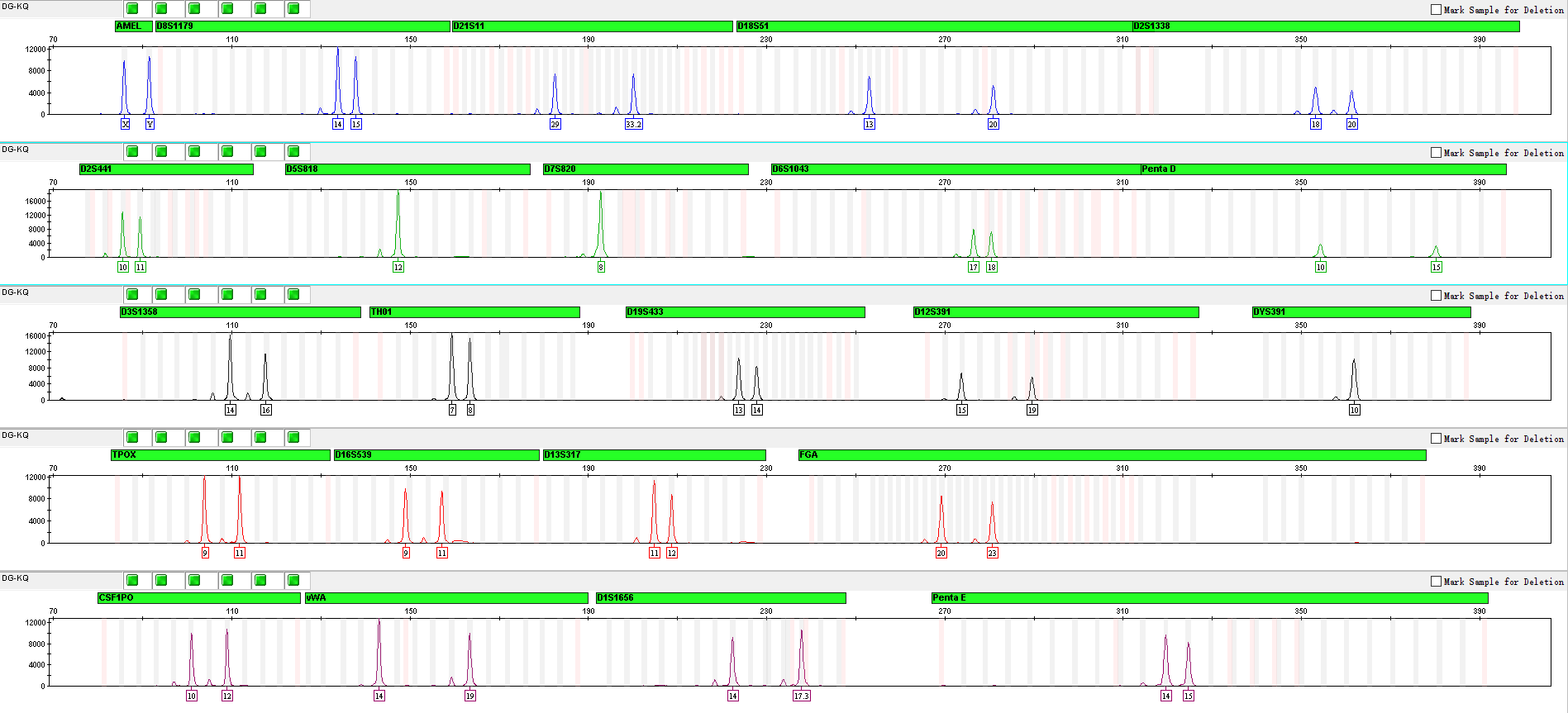


1. Costal cartilage bone:


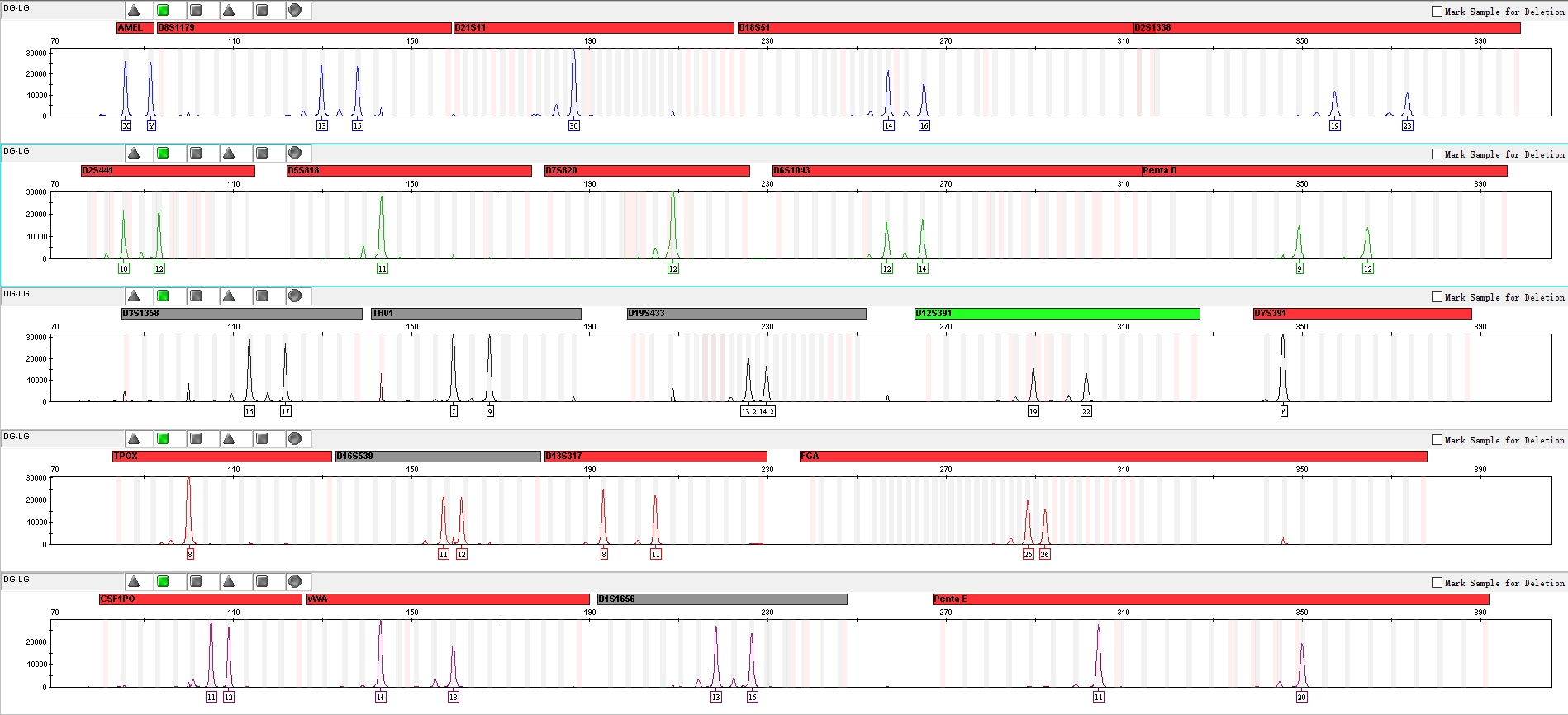


1. Semen:


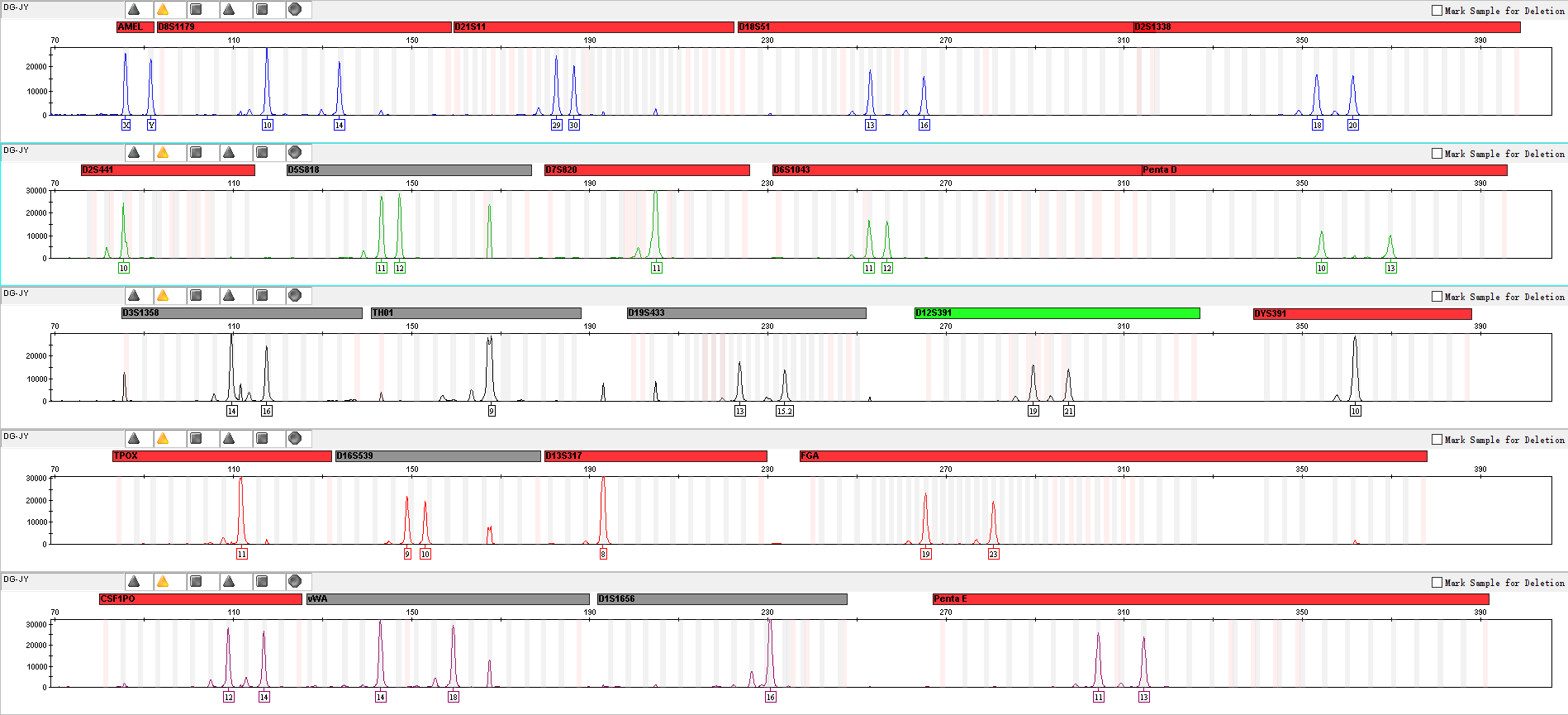


1. Blood specimen extracted by ML- DNA Extraction Kit (Bokun Biotech):


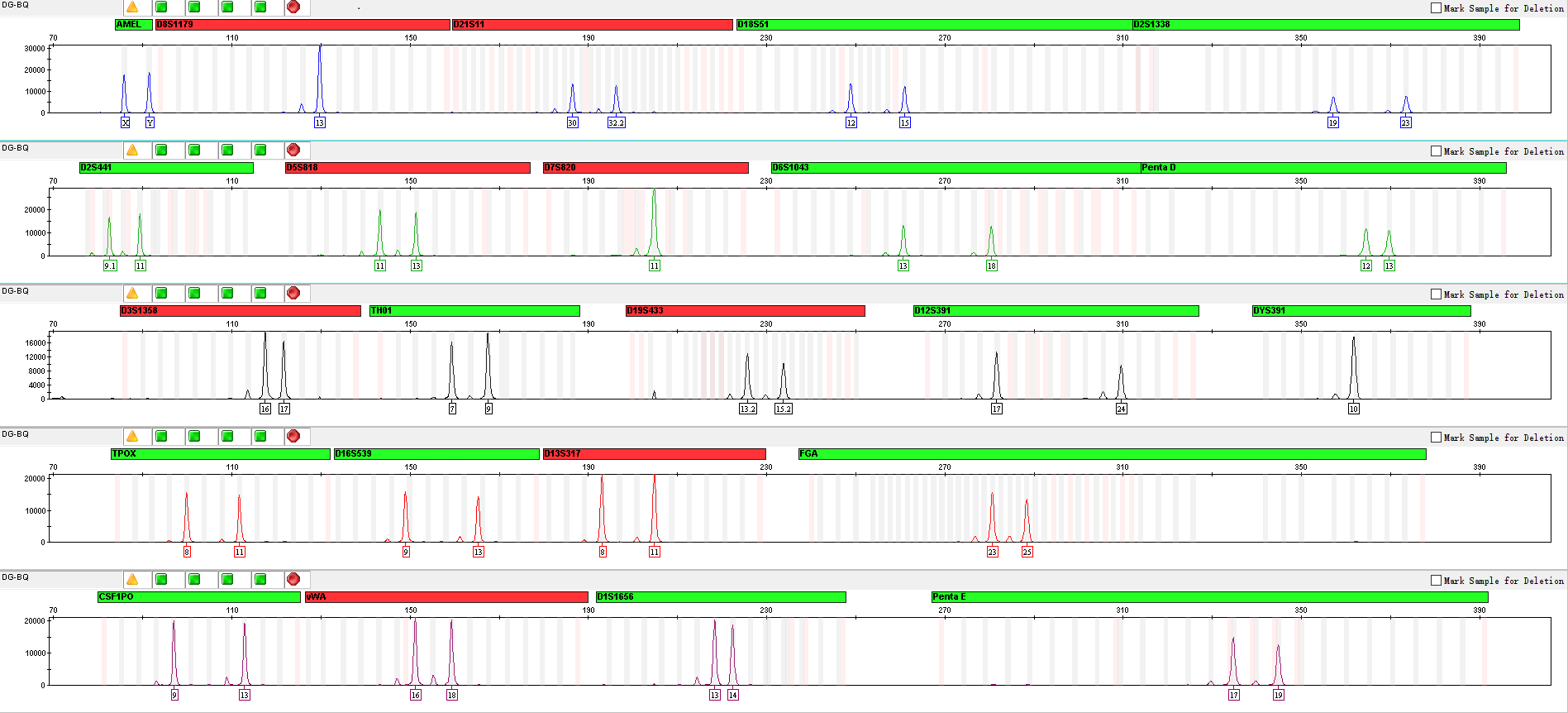


1. Blood specimen extracted by QIAamp DNA Micro Kit:


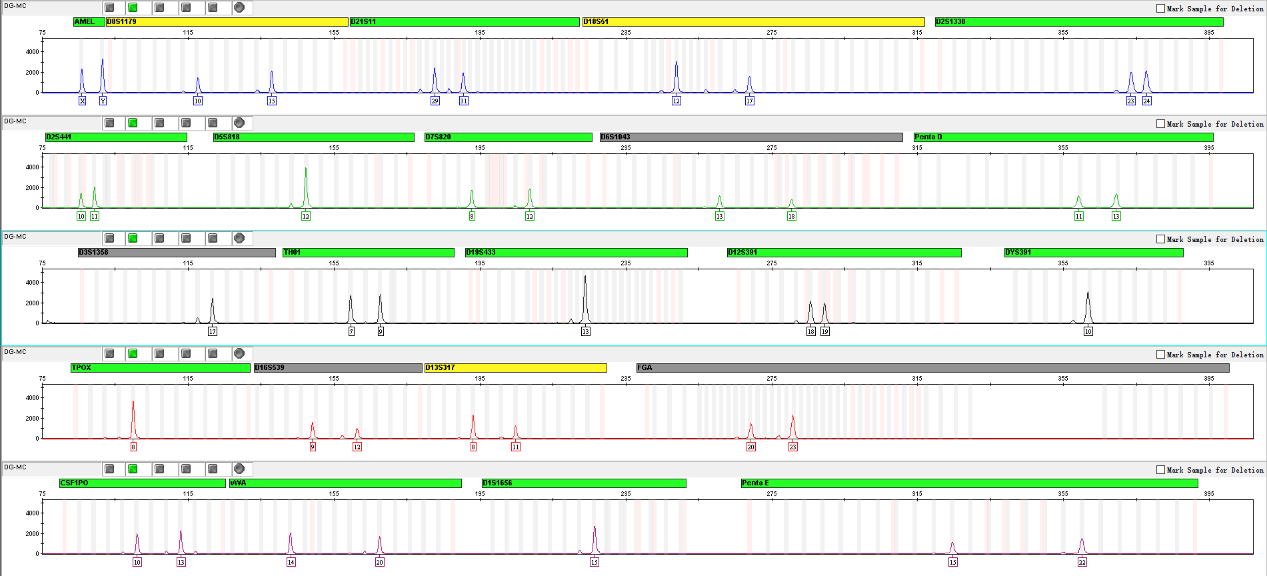


1. Blood specimen extracted by OptiPure Blood DNA (61E):


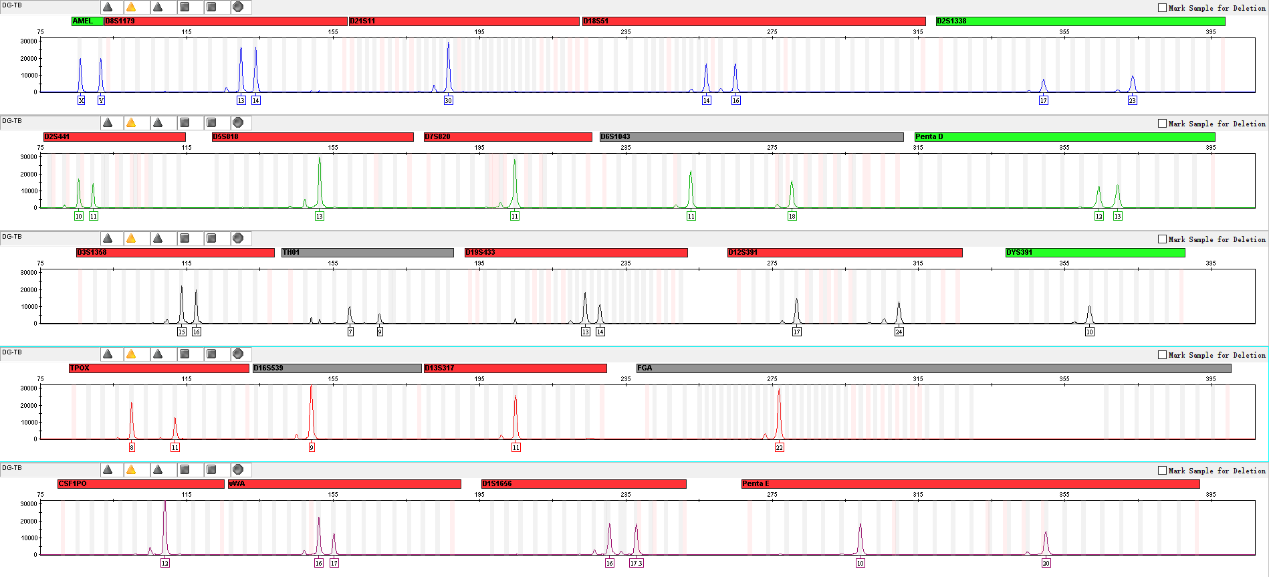


1. Blood specimen extracted by Chelex-100:


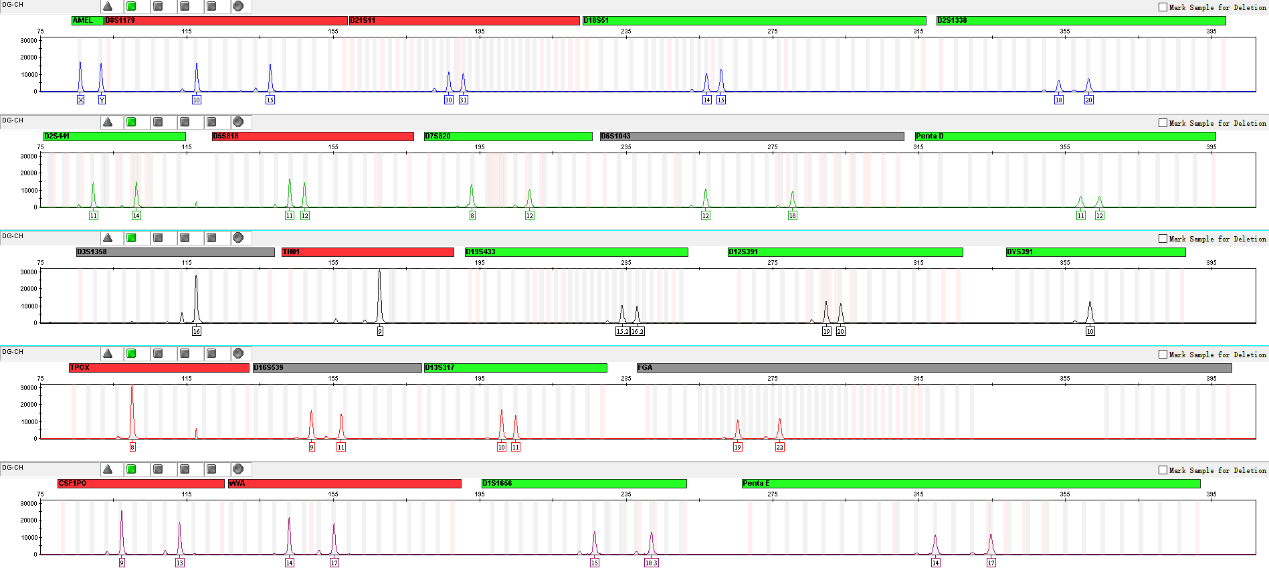


Supplemental Fig. S14. Profiles of ten case samples.


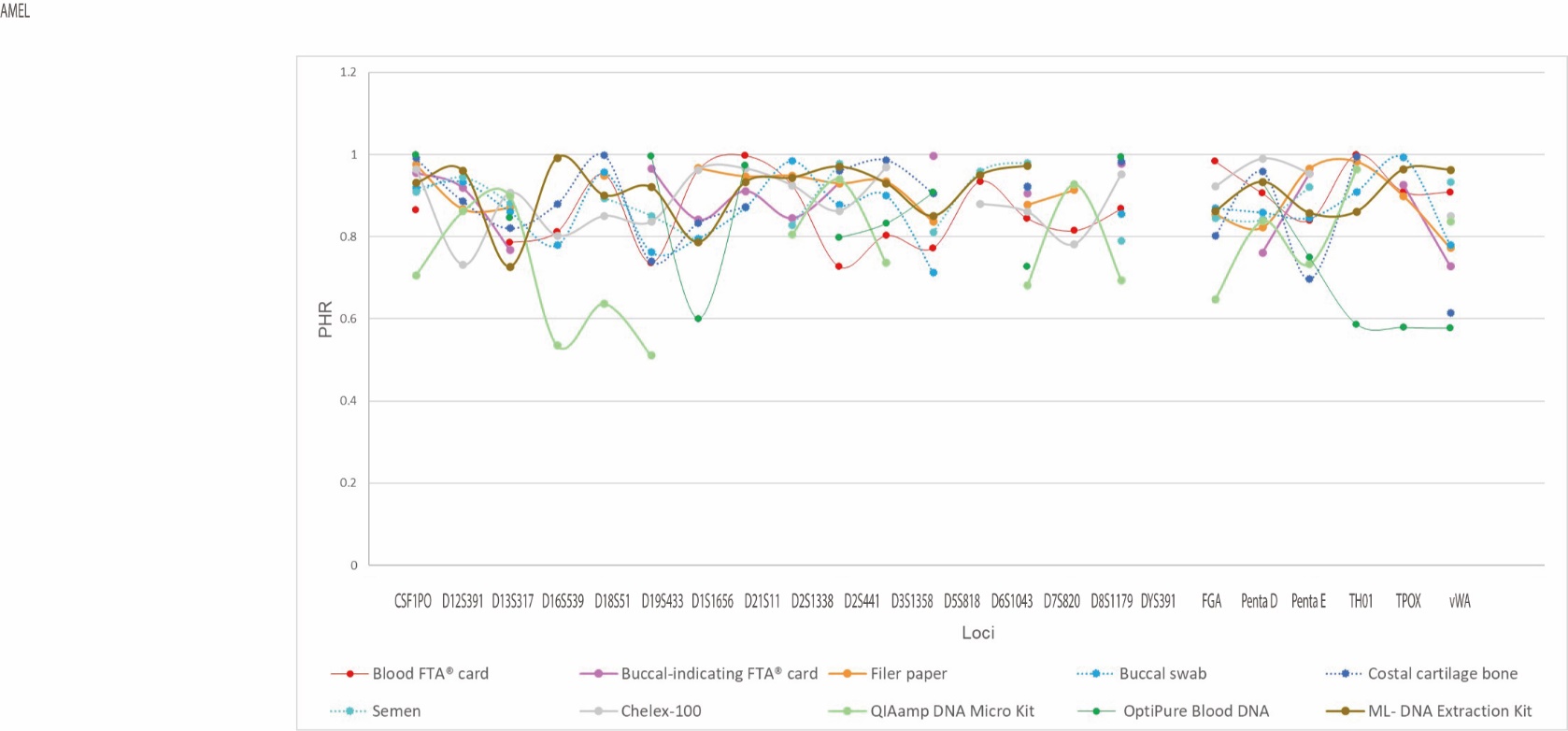


Supplemental Fig. S15. Intra-locus balance for ten case samples.


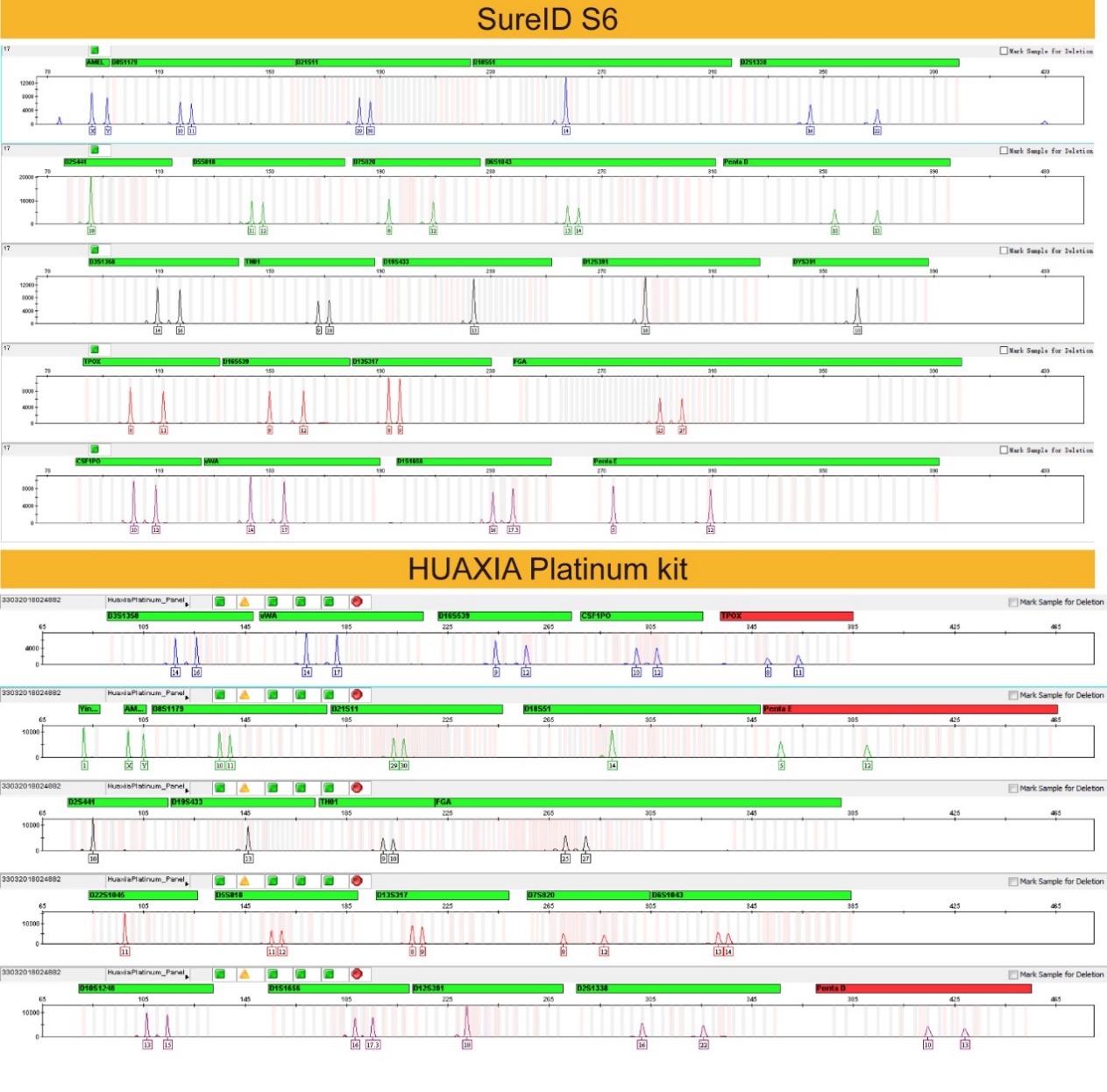


Supplemental Fig. S16. Concordance study tested between SureID^®^ S6 system and HUAXIA Platinum kit for Sample 2.

## Supplementary Tables (S1-S3)

Supplementary Table S1. Basic information about the 23 loci involved in the SureID^®^ S6 system.

| Loci | Position | Range (bp) | Ladder | genotype of 9948 | Color |
| --- | --- | --- | --- | --- | --- |
| AMEL | Xp22,Y:p112 | 84-92.5 | X, Y | X, Y | Blue |
| D8S1179 | 8q24.13 | 93-159 | 5, 6, 7, 8, 9, 10, 11, 12, 13, 14, 15, 16, 17, 18, 19 | 12, 13 | Blue |
| D21S11 | 21q21.1 | 159.5-222.5 | 24, 24.2, 25, 26, 27, 28, 28.2, 29, 29.2, 30, 30.2, 31, 31.2, 32, 32.2, 33, 33.2, 34, 34.2, 35, 35.2, 36, 37, 38 | 29, 30 | Blue |
| D18S51 | 18q21.33 | 223.5-317 | 7, 8, 9, 10, 10.2, 11, 12, 12.2, 13, 13.2, 14, 15, 16, 17, 18, 19, 20, 21, 22, 23, 24, 25, 26, 27, 28 | 15, 18 | Blue |
| D2S1338 | 2q35 | 320-399 | 11, 12, 13, 14, 15, 16, 17, 18, 19, 20, 21, 22, 23, 24, 25, 26, 27, 28 | 23 | Blue |
| D2S441 | 2p14 | 76-115 | 8, 9, 10, 11, 11.3, 12, 13, 14, 15, 16, 17 | 11, 12 | Green |
| D5S818 | 5q23.2 | 122-177 | 6, 7, 8, 9, 10, 11, 12, 13, 14, 15, 16, 17, 18 | 11, 13 | Green |
| D7S820 | 7q21.11 | 180-226 | 6, 7, 8, 9, 10, 11, 12, 13, 14, 15 | 11 | Green |
| D6S1043 | 6q15 | 228-311 | 7, 8, 9, 10, 11, 12, 13, 14, 15, 16, 17, 18, 19, 20, 21, 22, 23 | 12 | Green |
| Penta D | 21q22.3 | 314-396 | 2.2, 3.2, 5, 6, 7, 8, 9, 10, 11, 12, 13, 14, 15, 16, 17 | 8, 12 | Green |
| D3S1358 | 3p21.31 | 85-139 | 9, 10, 11, 12, 13, 14, 15, 16, 17, 18, 19, 20 | 15, 17 | Yellow |
| TH01 | 11p15.5 | 141-188 | 4, 5, 6, 7, 8, 9, 9.3, 10, 11, 12, 13, 13.3 | 6, 9.3 | Yellow |
| D19S433 | 19q12 | 191-252 | 6, 7, 8, 9, 10, 11, 12, 12.2, 13, 13.2, 14, 14.2, 15, 15.2, 16, 16.2, 17, 17.2, 18.2, 19.2 | 13, 14 | Yellow |
| D12S391 | 12p12 | 263-327 | 14, 15, 16, 17, 18, 19, 20, 21, 22, 23, 24, 25, 26 | 18, 24 | Yellow |
| DYS391 | Yq11.21 | 339-388 | 5, 6, 7, 8, 9, 10, 11, 12, 13, 14, 15 | 10 | Yellow |
| TPOX | 2p25.3 | 83-132 | 5, 6, 7, 8, 9, 10, 11, 12, 13, 14, 15 | 8, 9 | Red |
| D16S539 | 16q24.1 | 133-179 | 5, 6, 7, 8, 9, 10, 11, 12, 13, 14, 15 | 11 | Red |
| D13S317 | 13q31.1 | 180-230 | 5, 6, 7, 8, 9, 10, 11, 12, 13, 14, 15, 16 | 11 | Red |
| FGA | 4q28 | 238-400 | 13, 14, 15, 16, 16.2, 17, 17.2, 18, 18.2, 19, 19.2, 20, 20.2, 21, 21.2, 22, 22.2, 23, 23.2, 24, 24.2, 25, 25.2, 26, 27, 28, 29, 30, 31.2, 32.2, 33.2, 34.2, 43.2, 44.2, 45.2, 46.2, 47.2, 48.2, 49.2, 50.2 | 24, 26 | Red |
| CSF1PO | 5q33.1 | 80-125.5 | 6, 7, 8, 9, 10, 11, 12, 13, 14, 15 | 10, 11 | Purple |
| vWA | 12p13.31 | 126.5-190 | 10, 11, 12, 13, 14, 15, 16, 17, 18, 19, 20, 21, 22, 23, 24 | 17 | Purple |
| D1S1656 | 1q42 | 196-252 | 8, 9, 10, 11, 12, 13, 14, 15, 15.3, 16, 16.3, 17, 17.3, 18.3, 19.3, 20.3 | 14, 17 | Purple |
| Penta E | 15q26.2 | 267-392 | 5, 6, 7, 8, 9, 10, 11, 12, 13, 14, 15, 16, 17, 18, 19, 20, 21, 22, 23, 24, 25, 26, 27 | 11 | Purple |

Supplementary Table S2. The forensic parameters of 21 autosomal-STR loci in the Han population residing in Zhejiang province, China.

| Loci | MP | PIC | PD | PE | TPI | H*e* | *P* |
| --- | --- | --- | --- | --- | --- | --- | --- |
| D8S1179 | 0.0406 | 0.8375 | 0.9594 | 0.7447 | 4.0000 | 0.8750 | 0.1747 |
| D21S11 | 0.0588 | 0.7953 | 0.9412 | 0.6526 | 2.9126 | 0.8283 | 0.5177 |
| D18S51 | 0.0377 | 0.8451 | 0.9623 | 0.7080 | 3.4884 | 0.8567 | 0.7571 |
| D2S1338 | 0.0332 | 0.8556 | 0.9668 | 0.7380 | 3.8961 | 0.8717 | 0.9071 |
| D2S441 | 0.0853 | 0.7447 | 0.9147 | 0.5715 | 2.3256 | 0.7850 | 0.6382 |
| D5S818 | 0.0842 | 0.7456 | 0.9158 | 0.6021 | 2.5210 | 0.8017 | 0.1902 |
| D7S820 | 0.0916 | 0.7302 | 0.9084 | 0.5299 | 2.0979 | 0.7617 | 0.8453 |
| D6S1043 | 0.0305 | 0.8581 | 0.9695 | 0.6916 | 3.2967 | 0.8483 | 0.0749 |
| PentaD | 0.0544 | 0.7998 | 0.9456 | 0.6272 | 2.7027 | 0.8150 | 0.6485 |
| D3S1358 | 0.1184 | 0.6835 | 0.8816 | 0.4283 | 1.6667 | 0.7000 | 0.0855 |
| TH01 | 0.1587 | 0.6221 | 0.8413 | 0.3691 | 1.4706 | 0.6600 | 0.7376 |
| D19S433 | 0.0702 | 0.7797 | 0.9298 | 0.6430 | 2.8302 | 0.8233 | 0.2481 |
| D12S391 | 0.0406 | 0.8312 | 0.9594 | 0.6457 | 2.8524 | 0.8247 | 0.0887 |
| TPOX | 0.2117 | 0.5495 | 0.7883 | 0.2791 | 1.2195 | 0.5900 | 0.2985 |
| D16S539 | 0.0788 | 0.7524 | 0.9212 | 0.5776 | 2.3622 | 0.7883 | 0.8832 |
| D13S317 | 0.0713 | 0.7687 | 0.9287 | 0.5565 | 2.2388 | 0.7767 | 0.1782 |
| FGA | 0.0334 | 0.8518 | 0.9666 | 0.7213 | 3.6585 | 0.8633 | 0.8237 |
| CSF1PO | 0.1109 | 0.6983 | 0.8891 | 0.4734 | 1.8405 | 0.7283 | 0.4545 |
| vWA | 0.0689 | 0.7768 | 0.9311 | 0.6272 | 2.7027 | 0.8150 | 0.5798 |
| D1S1656 | 0.0452 | 0.8209 | 0.9548 | 0.5960 | 2.4793 | 0.7983 | 0.0063 |
| PentaE | 0.0146 | 0.9067 | 0.9854 | 0.8140 | 5.5000 | 0.9091 | 0.6749 |

** MP, match probability; PIC, polymorphic information content; PD, power of discrimination; PE, power of exclusion; TPI, typical paternity index; He, expected heterozygosity; p, probability of Hardy-Weinberg equilibrium.*

Supplementary Table S3. Heterozygote balance studies performed to compare the performance of SureID^®^ S6 system and HUAXIA Platinum kit.

| Loci | Sample 1  SureID^®^ S6 | | Sample 1  HUAXIA Platinum kit | | Sample 2  SureID® S6 | | Sample 2  HUAXIA Platinum kit | |
| --- | --- | --- | --- | --- | --- | --- | --- | --- |
|  | Genotype | PHR | Genotype | PHR | Genotype | PHR | Genotype | PHR |
| AMEL | X/Y | 0.9143 | X/Y | 0.9155 | X/Y | 0.8381 | X/Y | 0.8356 |
| D8S1179 | 10/14 | 0.9159 | 10/14 | 0.9730 | 10/11 | 0.9754 | 10/11 | 0.5589 |
| D21S11 | 30/31.2 | 0.9416 | 30/31.2 | 0.9621 | 29/30 | 0.2095 | 29/30 | 0.8258 |
| D18S51 | 14/16 | 0.9446 | 14/16 | 0.9913 | 14/14 | 0.5214 | 14/14 | 0.8121 |
| D2S1338 | 20/23 | 0.8787 | 20/23 | 0.8162 | 16/22 | 0.9383 | 16/22 | 0.9720 |
| D2S441 | 11/12 | 0.8655 | 11/12 | 0.9031 | 10/10 | 0.9206 | 10/10 | 0.8766 |
| D5S818 | 11/12 | 0.8726 | 11/12 | 0.9069 | 11/12 | 0.8837 | 11/12 | 0.4212 |
| D7S820 | 8/10 | 0.9559 | 8/10 | 0.9166 | 8/12 | 0.5644 | 8/12 | 0.7400 |
| D6S1043 | 12/13 | 0.9252 | 12/13 | 0.8040 | 13/14 | 0.9261 | 13/14 | 0.6395 |
| Penta D | 10/11 | 0.8495 | 10/11 | 0.9497 | 10/13 | 0.9744 | 10/13 | 0.3321 |
| D3S1358 | 15/17 | 0.9894 | 15/17 | 0.9665 | 14/16 | 0.8816 | 14/16 | 0.9632 |
| TH01 | 7/9 | 0.8493 | 7/9 | 0.8908 | 9/10 | 0.8961 | 9/10 | 0.5929 |
| D19S433 | 13/15.2 | 0.9922 | 13/15.2 | 0.9105 | 13/13 | 0.9608 | 13/13 | 0.9488 |
| D12S391 | 18/22 | 0.8137 | 18/22 | 0.8832 | 18/18 | 0.7991 | 18/18 | 0.6995 |
| DYS391 | 10/10 | / | / | / | 10/10 | / | / | / |
| TPOX | 9/11 | 0.9170 | / | / | 8/11 | 0.9206 | / | / |
| D16S539 | 9/10 | 0.8952 | 9/10 | 0.9084 | 9/12 | 0.5956 | 9/12 | 0.4330 |
| D13S317 | 8/11 | 0.8727 | 8/11 | 0.9721 | 8/9 | 0.7225 | 8/9 | 0.7046 |
| FGA | 23/25 | 0.9142 | 23/25 | 0.9366 | 25/27 | 0.9064 | 25/27 | 0.9910 |
| CSF1PO | 11/12 | 0.8872 | 11/12 | 0.7740 | 10/12 | 0.9089 | 10/12 | 0.9744 |
| vWA | 14/16 | 0.9638 | / | / | 14/17 | 0.9900 | / | / |
| D1S1656 | 14/16 | 0.9522 | 14/16 | 0.9764 | 16/17.3 | 0.8392 | 16/17.3 | 0.4655 |
| Penta E | 16/18 | 0.9286 | 16/18 | 0.8950 | 5/12 | 0.9048 | 5/12 | 0.2864 |
